# A qualitative research synthesis of contextual factors contributing to female overweight and obesity over the life course in sub-Saharan Africa

**DOI:** 10.1371/journal.pone.0224612

**Published:** 2019-11-04

**Authors:** Ifeoma D. Ozodiegwu, Mary Ann Littleton, Christian Nwabueze, Oluwaseun Famojuro, Megan Quinn, Richard Wallace, Hadii M. Mamudu

**Affiliations:** 1 Institute for Global Health, Feinberg School of Medicine, Northwestern University, Chicago, Illinois, United States of America; 2 Department of Community and Behavioral Health, East Tennessee State University, Johnson City, Tennessee, United States of America; 3 Department of Biostatistics and Epidemiology, East Tennessee State University, Johnson City, Tennessee, United States of America; 4 Quillen College of Medicine Library, East Tennessee State University, Johnson City, Tennessee, United States of America; 5 Department of Health Services Management and Policy, East Tennessee State University, Johnson City, Tennessee, United States of America; University of Lincoln, UNITED KINGDOM

## Abstract

**Objective:**

Adult women are disproportionately affected by overweight and obesity in Sub-Saharan African (SSA) countries. Existing evidence on the sociocultural context remains unconsolidated. In this qualitative research synthesis, we aggregate research literature on contextual factors that potentially predispose adult women and adolescent girls to overweight and obesity to inform research, policies and programs over the life course.

**Methods:**

PubMed, CINAHL, PsychInfo, ProQuest Central, EMBASE, and Web of Science were searched to locate qualitative research articles conducted in SSA countries beginning in the year 2000. After assessment for eligibility and critical appraisal, 17 studies were included in the synthesis. Textual data and quotes were synthesized using meta-aggregation methods proposed by the Joanna Briggs Institute.

**Results:**

The synthesized studies were conducted in South Africa, Ghana, Kenya and Botswana. The three overarching themes across these studies were body size and shape ideals, barriers to healthy eating, and barriers to physical activity, with cultural and social factors as cross-cutting influences within the major themes. Culturally, the supposedly ideal African woman was expected to be overweight or obese, and voluptuous, and this was associated with their identity. Although being overweight or obese was not acceptable to adolescent girls, they desired to be voluptuous. Healthy food choices among women and adolescent girls were hampered by several factors including affordability of nutritious foods and peer victimization. Both adult women and adolescent girls experienced ageism as a barrier to physical activity.

**Significance:**

This is the first qualitative research synthesis to amplify the voices of women and girls in SSA countries highlighting the challenges they face in maintaining a healthy body weight. Sociocultural, institutional and peer-related factors were powerful forces shaping body size preferences, food choices and participation in physical activity. Our study findings provide insights for the design of contextually appropriate obesity prevention interventions and lay the foundation for further research studies.

## Introduction

The growing prevalence of overweight and obesity among Sub-Saharan African (SSA) women is alarming. From 1975 to 2016, age-standardized adult female overweight prevalence increased by 24%, to 39%, while obesity prevalence increased by 12%, to 15%; whereas, for adult males, age-standardized overweight prevalence in the same time period increased only by 15%, to 23% and obesity prevalence by 5%, to 6% [1]. Although biological differences between males and females may partly explain the disproportionate prevalence of overweight and obesity among SSA women [2], extant evidence suggests that the sociocultural context matters. For instance, cultural ideals that regard overweight and obese women as beautiful influence cultural practices such as force-feeding, performed to hasten physical development and marriage in adolescent girls [3]. In an analysis of the 2001 Demographic and Health Survey from Mauritania, the authors found that obese women reported a greater prevalence of force-feeding [4].

Identifying contextual factors contributing to increased female adiposity in SSA is essential to supporting efforts to halt the rise in obesity, one of the global voluntary targets outlined in the World Health Organization (WHO) Global Action Plan for the Prevention and Control of Non-communicable Diseases (NCDs) [5]. Additionally, given that overweight and obesity are associated with various chronic health conditions, and premature mortality [6,7], achieving progress for obesity-related targets will likely feed into the accomplishment of the Sustainable Development Goal 3.4, which is to reduce premature mortality from NCDs by one-third [8].

One of the overarching principles of the aforementioned WHO Global Action Plan is the recognition that a life-course approach to the prevention and control of NCDs is crucial [5]. Adopting a life-course approach permits the identification of potential adiposity risk factors and prevention needs at multiple life stages. This is consequential because adolescence is potentially a critical period for targeting interventions to reduce the likelihood of later life adiposity and NCDs. A systematic review and meta-analysis of studies suggests that roughly 80% of obese adolescents remain obese in early adulthood and 70% after age 30 [9]. This indicates that efforts to decrease the burden of adult female overweight and obesity must also target contributing factors in adolescents. A systematic presentation of overweight and obesity determinants in adolescent girls and adult women in SSA will assist in the identification of similarities and differences in their lived experiences to inform research, programs and policies.

By using a qualitative research synthesis approach, also known as meta-aggregation, existing evidence is consolidated and concisely presented increasing the accessibility of current findings to the research community and policymakers. Similar methods have been employed in other studies, and the findings have informed public health prevention activities. For instance, a Cochrane Systematic Review used a qualitative research synthesis approach to assess how context affects the implementation of lay health worker programs for improving the delivery of maternal and child health care [10]. The results of this review were used by the WHO to develop guidelines to inform implementation in various contexts [11,12].

Qualitative evidence is especially critical in overweight and obesity prevention at this time because a recent evaluation study suggested that intervention policies, often touted as the most-effective obesity prevention measure, were falling short of expectations in high-income countries [13]. Insufficient consideration of contextual factors in designing and implementing policy solutions may account for this surprising finding. As SSA countries progress towards embracing policy interventions for the prevention of overweight and obesity, other metabolic conditions, and NCDs in general, there is need for aggregated evidence to inform its design. Therefore, this research synthesis aimed to consolidate and summarize existing literature on contextual factors associated with overweight and obesity, food choices, and physical activity among adult women and adolescent girls residing in sub-Saharan Africa. This study was guided by the following three questions:

1. What cultural and social norms are associated with overweight, obesity, poor food choices, and inadequate physical activity among adult African women and adolescent girls?
2. What knowledge, attitudes and perceptions contribute to overweight, obesity, poor food choices, and inadequate physical activity among adult African women and adolescent girls?
3. What institutional factors or policies are associated with overweight, obesity, poor food choices and inadequate physical activity among adult African women and adolescent girls?

## Methods

### Search strategy

An exhaustive search of six databases was conducted in PubMed, CINAHL, PsychInfo, ProQuest Central, EMBASE, Web of Science using a combination of search terms including “overweight”, “obesity”, “cultural norms”, “social norms”, “Africa”, between November 18^th^, 2018 and January 13^th^, 2019 to identify journal articles and dissertations related to the study aims. Search findings were subsequently filtered using terms such as “qualitative” and restricted to studies conducted from the year 2000 to ensure that they were relevant to the current time period. An example of a search strategy used in PubMed is:

((("Obesity"[Mesh]) OR "Overweight"[Mesh]) AND "Africa South of the Sahara"[Mesh]) Filters: Publication date from 2000/01/01
(("Nutritional Status"[Mesh] AND ("2000/01/01"[PDat]: "3000/12/31"[PDat]))) AND "Africa South of the Sahara"[Mesh] Filters: Publication date from 2000/01/01

Additional search terms can be viewed in the Appendix. With the exception of the records from ProQuest Central, all the records found using the search terms were included in the screening process. The search terms and filters used in ProQuest Central–search terms: “physical activity or food or overweight or obesity and qualitative”, filters: Scholarly journals or dissertations/theses, 2001-01-01–2019-01-13, Africa—resulted in thousands of unrelated articles in later pages. We tried to use narrow search terms, but this excluded important articles we already identified in other databases. Therefore, we limited the records included in the screening to the first five pages, which contained 100 records. This was because these records contained related articles that we found in other databases as well as new related records.

### Inclusion criteria

Only primary qualitative studies written in the English language were included in this study. Using the PICo mnemonic proposed by the Joanna Briggs Institute (JBI) [14], the inclusion criteria were outlined as follows:

1. Population: Healthy adolescent and adult females without regard for educational status, social status and ethnicity. Adolescents were defined as individuals aged ten to nineteen in accordance with the WHO standards [15]. Studies with male participants were excluded except when the results of the study were reported separately for females, or when the male participants reported their perceptions of factors associated with female overweight and obesity.
2. Context: Studies must be conducted within SSA countries.
3. Phenomena of Interest: Cultural and social norms, knowledge, attitudes, perceptions, and institutional factors associated with overweight and obesity, poor food choices, and inadequate physical activity.

### Screening

**Fig 1:**
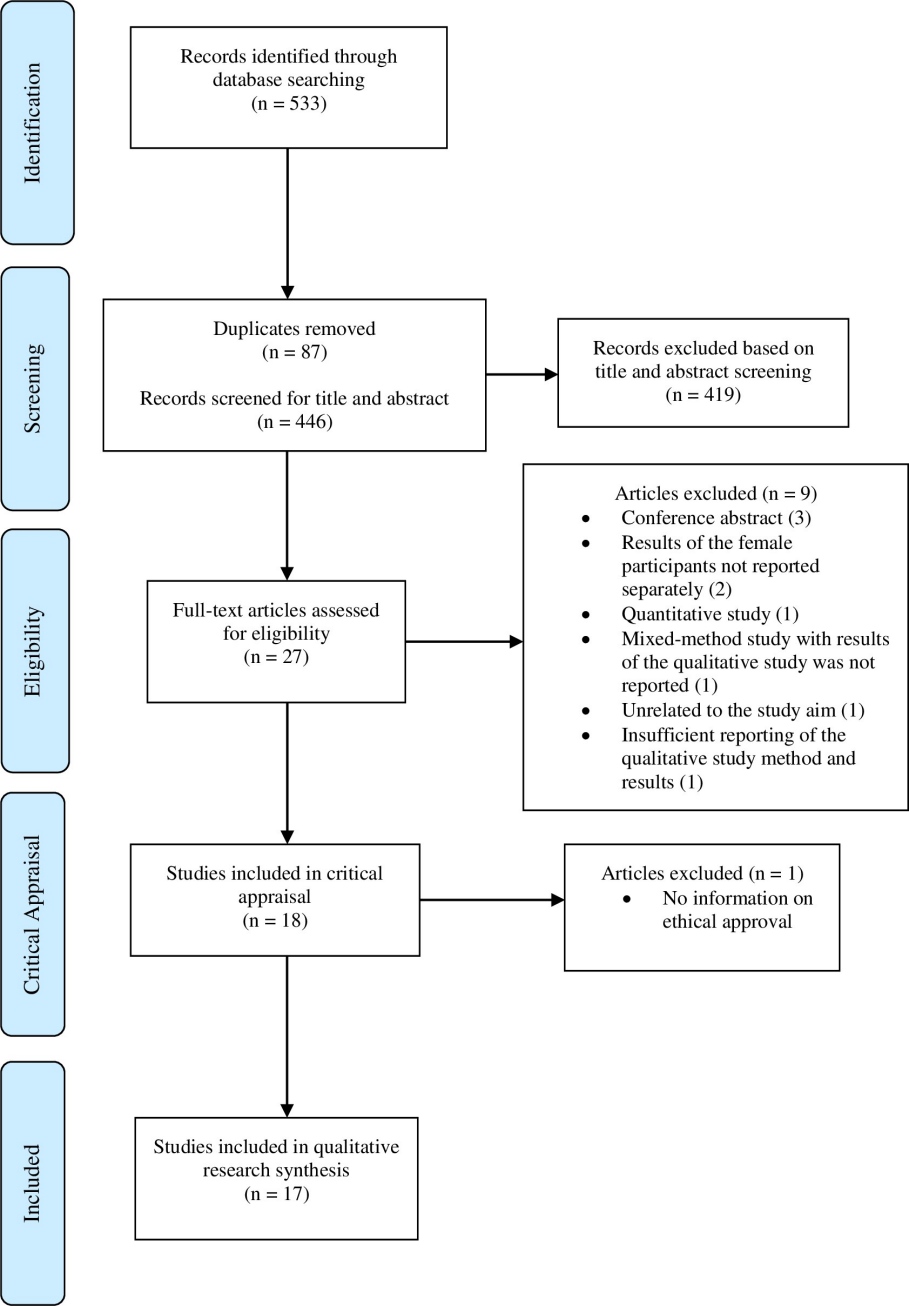
PRISMA flow diagram.

The database search yielded 533 records. After the removal of duplicates and any studies conducted prior to 2000 (n = 87), a total of 446 records remained. Two reviewers (IDO and OF) evaluated the titles and abstracts against the inclusion criteria resulting in the exclusion of 419 records. The full text of the remaining 27 articles were independently evaluated for eligibility by two reviewers (IDO and CN) using the inclusion criteria. A total of 9 articles were excluded leaving 18 articles for inclusion in the critical appraisal. The reasons for excluding the aforementioned articles are reflected in Fig 1, the PRISMA flow diagram.

### Critical appraisal

Two reviewers (IDO and CN) independently evaluated the methodologic appropriateness, quality and rigor of the remaining 18 articles using the Critical Appraisal Skills Program (CASP) tool for Qualitative research. The tool provides 10 questions for assessing the following: the clarity of the research aims and findings, the appropriateness of the methodology, recruitment, data collection, data analysis, and ethical procedures, and researcher’s reflexivity. However, due to the dependence of most of the appraisal criteria on journal reporting requirements and page limits, it was decided that articles will only be excluded if the response was negative for the following questions in the CASP tool:

1. Was there a clear statement of the aims of the research?
2. Is the qualitative methodology appropriate?
3. Have ethical issues been taken into consideration?
4. Is there a clear statement of findings?

Based on the critical appraisal exclusion criteria, one article was eliminated because it lacked information on ethical approval for their studies. Consequently, 17 articles were utilized for the qualitative evidence synthesis. The reporting of this review followed the principles recommended in the Preferred Reporting in Systematic Reviews and Meta-Analyses (PRISMA) statement (16).

### Data extraction

A pre-piloted standard form was used by one reviewer (IDO) to extract the key study details, the demographic characteristics of the participants (where available), and the main study findings for all 17 included studies. Using the method outlined by JBI [14], we considered findings to be demographic information included in the article and results of a thematic analysis. We only selected study findings corresponding to the focus of our synthesis and with an unequivocal level of plausibility, defined by the JBI as findings that cannot be disputed because they were accompanied by illustrative quotes and/or text that were congruent with the study [14]. Repeated readings of the extraction were undertaken by two reviewers (IDO and CN) and agreement on the validity of the extraction by a single reviewer (IDO) was reached with a second reviewer (CN) after comparison with the material in the included articles. In accordance with the focus of this research synthesis, pertinent study findings extracted from each article were specific to cultural and social norms and perceived behavioral and ecological risk factors reported by study participants that potentially predispose them to overweight and obesity, inadequate physical activity and poor food choices.

### Data synthesis

A thematic analysis framework according to the methods proposed by the JBI for meta-aggregation was used to summarize the findings of this synthesis [14], While the method has been previously described [14], a brief summary is provided. First, findings are categorized based on criteria defined by the researcher. Finally, one or more themes are developed by synthesizing findings within each category.

In the data synthesis for this study, all decisions were arrived at through a consensus process by two of the authors (IDO and CN). After repeated readings of the extracted data, findings from each study were categorized on the basis of concept similarity and restated in a summarized fashion. Subsequently, themes were identified, and related findings were further summarized by re-conceptualizing the information within each category. An example of the process is provided in Table 1. Lastly, major and sub-themes were finalized and reported with supporting statements and quotes.

**Table 1:** Example of the data synthesis process.

Finding with citation	Category label–Body shape and size ideals	Synthesized findings
Among the women, it was common opinion that women are required (by culture) to be overweight. [16]	There was a cultural expectation for women to be overweight.	There are cultural expectations for adult SSA women to have large and voluptuous bodies. Notably, men perceived the ideal body weight for women to be overweight or obese, whereas women perceived that of men to be normal weight or overweight. Women who were larger were regarded as more attractive, wealthy, and respectable. Women also had to be voluptuous to be viewed as beautiful.
Men: Larger silhouettes size 7–15 (overweight/obese categories) chosen as ideal normal size for a woman…., Women: ….less than size 13 (overweight or normal) for man [16]	Men indicated that the ideal body size for women was overweight/obese while the ideal for men as indicated by women was overweight or normal weight.	
This group was assured that being overweight is linked to high blood pressure and diabetes. However, they also said it was desirable to be big, because as a woman you look dignified, and people can see that you have enough money to feed yourself and your family, and that “traditional-looking” women look beautiful when they have big hips. [17]	Being overweight was regarded as desirable because community members viewed overweight women as wealthy and respectable. Women also had to be overweight and voluptuous to be regarded as beautiful.	

## Results

### Description of the included studies

A total of 17 studies were included in this qualitative research synthesis. Thirteen of them were primarily exploratory qualitative studies while four studies combined exploratory qualitative methods with quantitative methods (mixed-methods studies). Thirteen studies were conducted in South Africa [16,17, 18–25, 26–28], two studies in Ghana [29,30] and one study in Botswana [31] and one in Kenya [32]. The Botswana and Kenya studies were PhD dissertations while the rest were journal articles. Focus group discussions and semi-structured in-depth interviews were the primary methods used for data collection and the thematic analysis method was used for textual analyses. Sample sizes varied according to the data collection methods ranging from eight participants in in-depth interviews to 60 participants in focus group discussions. Although two studies were based on the same sample, both were included in this synthesis because they provided differing levels of detail on the findings of the study [25,27]. Table 2 presents the characteristics of each included study and a summary of its key findings related to the research synthesis’ aims.

**Table 2:** Description of included studies.

Author (Year)/Country/City or Town	Study Type/Data Collection/Data Analysis	Sample size†	Demographics†	Summary of key findings related to synthesis
Bodiba (2008)/South Africa [18]	Mixed methods study/Focus group/Audio-recorded/ Thematic analysis	9 females	Adolescent (17–19 years) first year university students	• Larger body sizes were regarded as part of the African identity. • Overweight people had no room to participate in sporting activities and often experience discrimination. • A curvy figure (having a smaller waist) was perceived more positively even when one was overweight.
Brown (2014)/Botswana/Gaborone [31]	Qualitative study/Focus group/Note-taking/Thematic analysis	12 adolescent male and female focus groups, and 3 adult male and female (parents) focus groups	Low and high SES adolescent (12–18 years) secondary school enrollees‡	• Decisions regarding physical activity, diet and obesity were influenced by time, place and company. • Being away from home created an opportunity to purchase sugar-sweetened drinks and fast foods. • Adolescent females indicated that their peers influenced their food choices and nonconformance to prevailing selections risked peer disapproval. • Parents agreed that peer influence played a role in adolescent female food choices. • Female study participants indicated that while they preferred a healthy body weight, they wanted to be voluptuous so that they “are able to wear nice clothes”. • Study participants reported that their family and community members viewed obese individuals as attractive, wealthy and strong. • Among the study participants, however, obesity was stigmatized. • Equally, being thin was negatively perceived. • Thinness and rapid weight loss were associated with diseases such as HIV infection.
Draper (2015)/South Africa/Cape Town [19]	Qualitative study/Semi-structured focus groups/Thematic analysis	21 females	Low SES urban adults (24–51 years)	• Being overweight or obese was acceptable and considered attractive by some participants because it protected them from judgement and criticism, and it was aligned with cultural expectations. • All study participants regarded obese people as being "too fat". • There were negative perceptions of slender individuals because thinness was associated with ill-health. • Individuals that lose weight for health reasons were also perceived negatively. • Highly processed foods, fatty foods and certain animal products were cheap, but vegetables were expensive. • Overconsumption of high energy staple foods was also implicated in weight gain. • Older community members received criticism when they engaged in exercise. • Lack of facilities and the financial ability to join a gym were identified as barriers to physical activity.
Hunter-Adams (2016)/South Africa/Cape Town [20]	Qualitative study/In-depth interviews/Focus groups/Audio-recorded/thematic analysis	23 female participants for the in-depth interviews and 48 female and male participants in the focus groups	Inner city migrant adult women (20–40 years) and men (20 –≥ 60 years)	• The migrants in the study from Congo and Zimbabwe generally desired traditional foods from their home country such as leafy greens, ground nuts and dried fish but were unable to access such foods in their new location because they were expensive and required forethought. • Somali migrants expressed that they experienced difficulty accessing local foods from their home country with comparable quality to what was found in Somalia. • Female study participants reported that they ate fast foods during pregnancy to satisfy cravings and also because it was the only time that their significant other was willing to cover fast food-related expenses. • Fast foods were used to compensate for the absence of traditional foods from their home countries. • Female study participants were encouraged to satisfy any cravings they had during pregnancy. • Fast foods were considered healthy because they helped to combat nausea. • The interviewees felt that the nutrition advice given to them at antenatal clinics did not account for their life situation.
Kinsman (2015)/South Africa/Agincourt [21]	Qualitative study/Focus group discussions/Audio recorded/Thematic analysis	51 adolescent girls, and 7 key informants (6 adult men and 1 adult woman)	Low SES rural adolescent girls (13–19 years), and sports teachers and youth leaders (21–44 years)	• Study participants reported that they were unwilling to engage in physical activity because they did not want to develop muscles. • Adolescent boys bullied muscular girls because muscularity was linked to poverty. The connection between muscularity and poverty arose from the belief that muscles were developed from engaging in physical activity typical among low SES individuals (e.g. walking to school and doing housework) in the community. • Study participants reported the presence of peer pressure to conform to slender and curvaceous body ideals. • Gender inequalities existed in opportunities to participate in sporting events. • Boys had better facilities and more funding for sporting events. • Key informants suggested that concerns about sexual violence and early initiation of sexual activity prevented parents from enrolling their girls in physical activity. • In general, physical activity was viewed as the domain of younger girls by peers and authority figures, and some respondents associated physical activity with health risks.
Matoti-Mvalo (2011)/South Africa/Cape Town [17]	Mixed methods study/Focus group/Audio-recorded/Note taking	20 females	Low SES urban adolescent girls and adults (18–65 years)	• HIV was highly prevalent in the community, and HIV-infected people experienced weight loss. Hence, some participants associated thinness with HIV. • Since HIV was a highly stigmatized condition, study participants resisted any association with it. • Therefore, being overweight was a preventive mechanism against stigma from HIV. • HIV-infected people were perceived more positively when they gained weight because community members believed that they were cured. • Being voluptuous was regarded as part of the culture and greater value was placed on women with this type of body frame.
Mugo (2016)/Kenya/ Subukia [32]	Qualitative study /Semi-structured in-depth interviews/Audio-recorded/Note-taking/Thematic analysis	8 females	Rural adults (20–45 years)	• One-half of the participants correctly described obesity. • Body size was an indicator for happiness, health, and attractiveness: big, round, wide and strong bodies were associated with positive attributes. • One participant reported desiring to gain more weight in order to feel more confident. • Participants viewed physical activity as a pursuit for young people. • The only form of physical activity that appeared acceptable was household-related duties.
Muzigaba (2014)/South Africa/ Western Cape Province [22]	Qualitative study/Focus groups/Audio-recorded/Thematic analysis	34 pregnant females	Low SES adolescents and adults (mean age = 25.6 years, SD = 5.2)	• Study participants were aware of the benefits of physical activity during pregnancy but had concerns about causing harm to their baby or themselves. • Pregnancy-related physical changes also prevented women from engaging in physical activity. • Other barriers to engaging in physical activity during pregnancy included lack of time due to household and work responsibilities, lack of facilities and family support, neighborhood safety, the perception that physical activity required a gym and high SES, and the absence of establishments that organize various forms of physical activity for pregnant women. • The interviewees indicated that access to informational booklets, physical activity programs in antenatal clinics, and guidance and permission from a healthcare professional would facilitate their engagement in physical activity.
Okop (2016)/South Africa/Langa (near Cape Town) [16]	Qualitative study/Semi-structured focus groups/Thematic analysis	36 females and 42 males	Low SES adults (35–70 years)	• There were cultural and normative expectations for women to be overweight. • Female participants had poor perception of their actual body size. • Weight-loss and thinness were perceived as a sign of ill health and stress while being overweight was a symbol of happiness and wealth. • Being overweight was acceptable and preferred as long as one did not exceed a certain threshold. Nonetheless, some younger women challenged the acceptance of overweight. • Normal weight and overweight women were dissatisfied with their body size and believed they would be more attractive if they gained more weight. • Obese people, but not overweight individuals, expressed a desire to lose weight. • Barriers to physical activity included the poor acceptance of structured physical activity/exercise and lack of facilities.
Phillips (2016)/South Africa/ Soweto [23]	Qualitative study/In-depth interviews/Audio-recorded/Thematic analysis	17 normal weight daughters, and 15 obese mothers	Adults (daughters’ mean age = 24.2 years, (SD = 0.04), mothers’ mean age = 53 years (SD = 4.9))	• Daughters were knowledgeable about healthy eating, but it was not a priority for several of them. • Weight gain was a sign of happiness and maturity. • For some mothers, purchasing fast food for their children was a means of providing a better upbringing for their children. For others, a better upbringing for their children meant giving them healthy food. • A possible protective factor for the daughters was that they ate smaller or normal sized meals. • Daughters indicated that barriers to exercise included paucity of time, money, and access to sports teams. • Participants viewed performing household duties as a form of exercise. • Mothers discouraged their daughters from participating in physical activity because they felt it was for younger children. • Additionally, mothers were of the view that exercising in the gym should not be prioritized by their daughters because it was a waste of money. • Mothers reported that they preferred large body sizes (US sizes 14–18) compared to daughters (US sizes 2–10).
Puoane (2010)/South Africa/Cape Town [24]	Mixed methods study/Focus group/Audio-recorded/Thematic analysis	60 females	Low SES urban primary and high school adolescents (10–18 years)	• Adiposity was associated with genetics, health, happiness, and wealth. • Thinness was associated with infectious diseases such as HIV, AIDS and or tuberculosis. • Obese girls held the opinion that obesity was preferable because it enables engagement in strenuous activities. • Some obese participants also indicated that obese people look more respectable and that obesity signifies good health. • In contrast, thin girls were of the view that fatness was linked to chronic conditions like diabetes and hypertension. • The authors found that interviewees were resistant to the idea of weight-loss to avoid stigma associated with HIV/AIDS or TB. • In general, weight-loss was linked to ill-health. • Thin girls reported that overweight or obese girls were often bullied.
Sedibe (2014)/South Africa/ Soweto [25]	Qualitative study/Duo semi-structured interview of adolescent and best friend/ Audio-recorded/Note taking/Thematic analysis	29 female pairs	Low SES urban adolescent & adult (mean = 18 years, SD = 1.2) final year secondary school enrollees	• Food choices at school depended on affordability, the type of food (traditional vs western diet), the level of enjoyment and satiety derived from the meal, and whether it could be shared with friends. • The interviewees preferred western diets and associated traditional meals with low SES. As such, they refrained from eating traditional meals at school because they viewed it as embarrassing. • Fruits sold in schools were of poor quality and expensive. • Fast foods or convenience foods were typically consumed at home during the weekends because it was inexpensive, accessible, and filling. Vegetables were not always available for home meals. • Caregivers often made the decision on what was consumed at home. • The majority of participants were sedentary. • Their communities lacked opportunities and support for recreational physical activity for young women and girls. • Safety and time were also barriers to participating in physical activity
Sedibe (2014)/South Africa/Agincourt [26]	Qualitative study/Duo in-depth interview/Audio-recorded/Thematic analysis	11 female pairs	Low SES rural adolescents (16–19 years)	• While the study participants rightfully pointed out that vegetables were a healthy food choice, it appeared that they had cloudy notions of what represented healthy food and its nutritional benefits. • Female caregivers had strong influence on the meals consumed by families. • Barriers to healthy eating included household poverty, affordability, and accessibility of healthy food, and peer and social influences. • Daily consumption of meat was viewed as a status symbol. • Study participants indicated a preference for junk food because it was filling, accessible, tasty, and more socially acceptable than consuming traditional foods. • Traditional foods were viewed as a sign of low SES. • Female students in their final year of secondary school were discouraged by school officials from participating in sports to prevent interference with their studies.
Tuakli-Wosornu (2014)/Ghana/Accra [30]	Mixed methods study/Focus groups/Semi-structured in-depth interviews /Thematic analyses	Unavailable	Urban older adult (58–71 years) females, fitness professionals (27–42 years), and clergy (40–72 years)	• Some study participants reported that they were unable to participate in physical activity due to concerns about time and self-injury. • Fitness coaches reported that older adult women were intimidated by gym equipment, male-dominated gyms, and the level of effort required to perform certain exercises. • There were no physical activity role models for older women. • Study participants were interested in the social elements of fitness and preferred group activities because of the opportunity for social interaction.
Tuoyire (2018)/Ghana/Accra and Tamale [29]	Qualitative study/Semi-structured interviews/Audio-recorded/Thematic analysis	36 females	Low & high SES urban adults (mean age = 33 years, SD = 9.2)	• Interviewees had a preference for larger body sizes that appeared to be in the overweight category, but this was difficult to assess from the study. • It was expected that the body size of the ideal African woman should be larger than that of western women. • The ideal body shape had to be voluptuous with a flat midsection. • The perceived ideal body shape was believed to look more attractive in outfits. • Thinness was associated with malnutrition. • Study participants encountered pressure from peers and family members to attain the ideal body size and this resulted in increased food consumption.
Voorend (2012)/South Africa/Soweto [27]	Qualitative study/Duo-interviewing technique/Audio-recorded	29 female pairs	Adolescent (mean age = 18 years (SD = 1.2)) final year secondary school enrollees	• Study participants preferred purchasing low nutritive value calorie dense foods from school because bringing lunch from home was considered embarrassing. • Food choices at school were determined by affordability, availability, waiting times, quality, and taste. • Fruits sold at school were deemed expensive. • Friends jointly made decisions about the type of food they consumed when they were together. • It often involved alternating between each other's food preferences or the decision was made by the person paying for the meal. • Food was often shared by friends.
Watson (2016)/South Africa/Soweto [28]	Qualitative study/Semi-structured interviews/Audio-recorded/Note-taking/Thematic analysis	13 pregnant females	Low and middle SES urban adolescents and adults (19–41 years)	• Interviewees were aware of the benefits of physical activity during pregnancy. • They assumed physical activity equated to only engaging in domestic-related physical activity. • Study participants were uncertain of the safety of different types of physical activity during pregnancy. • Some participants complained that the physiological and biological changes accompanying pregnancy, and the paucity of time and money to enroll in a gym prevented them from undertaking physical activity. • Participants also cited gaps in the provision of physical activity related information by healthcare practitioners, and the general unreceptive nature of health professionals to questions as additional barriers to embracing physical activity during pregnancy. • Participants indicated that they were not supported by family members to be physically active and were rather encouraged to be inactive during pregnancy. • Study participants suggested that the availability of community-based group exercise classes geared to the needs of pregnant women would encourage them to exercise.

†Inconsistencies in the description of demographic information were due to differences in the reported items in each article. Mean age and standard deviation were preferentially reported if they were available.

‡The demographic description for adults was not specified in the study.

### Data synthesis findings

Three major themes: (1) Body size and shape ideals, (2) barriers to healthy food choices, and (3) barriers to engaging in physical activity were identified from the thematic analysis. In the description of these themes, sub-themes within each overarching theme were presented for both adult women and adolescent girls.

### Body size and shape ideals

This major theme encompassed four sub-themes for adult SSA women. Factors that shaped preferred body sizes for women included culture and the social environment, the positive symbolism attached to large body sizes, and the association of thinness with HIV/AIDS infection. It was also found that body image dissatisfaction resulted from nonconformance to the preferred ideal and ignited a desire to gain more weight. While similarities existed between the experiences of adult women and adolescents, peer influence was a major determinant of body size and shape ideals among adolescents.

#### Culture and social environment

There were cultural expectations for adult SSA women to have large and voluptuous bodies [16,17,19,29]. An obese South African woman clearly described these cultural expectations: *"According to our values and culture*, *it is important for a woman to have a large body*. *It makes you to be respected [sic]*.*”* [16]. Notably, men perceived the ideal body size for women to be overweight or obese, whereas women perceived that of men to be normal weight or overweight [16]. Women internalized these expectations and perceived female fatness to be part of the African cultural identity [17,29]. Moreover, women encountered pressure from family and friends to attain normative cultural body sizes [29]. The ensuing quote by a normal weight Ghanaian woman exemplified this problem: *"My auntie has been asking me*, *‘why*, *don't your parents give you food to eat*? *Come to my house to eat’*. *She says as a woman I should eat and put on some flesh to look good*. *Every time she sees me she says the same thing*.*"* [29].

#### Positive symbolism of large body sizes

Women who were larger were regarded as more attractive [19]. Statements made by a South African focus group illustrates this phenomenon: "*Our culture says that we are supposed to be fat*. *You must have structure*, *[sic] you must be beautiful*. *In other words*, *our culture does not allow women to be thin*. *They say that someone who is fat is sexier*.” [19]. However, as underscored by the following quote attributed to a normal weight Ghanaian woman, there were thresholds to the ideal large body size: *"They want to see features as far as the Ghanaian woman is concerned*. *At least with some hips to keep the "coca-cola" shape kind [sic] at least to appear nice in your outfit*. *Yeah*, *I think that is it but not so fat"* [29]. Women also had to be voluptuous–large hips and bosoms, and small midsections–in order to be considered attractive [17,29]. Additionally, being overweight was regarded by some women as a sign of wealth, happiness, health, good nutrition and the absence of problems [16,32]. A South African overweight woman elucidated these perceptions: *"If a person is fat (overweight) we usually assume she is happy and has (lots of money)*. *It is evident that he/she eats nicely*, *and a lot*, *and not having problems* . . .” [16].

#### HIV/AIDS infection associated with thinness

The extant cultural ideals of beauty, the positive symbolism associated with overweight, and community experiences with morbidity from HIV/AIDS led to a perceived link between weight loss with ill-health and weight gain with recovery, which influenced preferred body sizes [16,17,19,29]. These ideas were reflected in the statements made in a focus group of South African women: “*These days*, *most people*, *especially the youth*, *are losing weight because of the HIV or AIDS*. *It is so abundant in this community*, *and every day you hear this one and that one have [sic] the disease*. *If you have AIDS and suddenly gain weight*, *people believe that you are well again*. *I know a girl who is fat and pretty*. *Now you cannot say she was sick*.*"* [17]. Women were resistant to losing weight to avoid being characterized as ill even though they were aware of the health conditions linked to being overweight [17,19]. A South African focus group was quoted as saying: “*She would not be happy [to lose weight] because in our community it would be perceived that there is something wrong with her*. *She would think that the community is looking at her*. *So*, *she would feel insecure*.” [19]. HIV, in particular, was heavily stigmatized and associated with risky sexual behaviors, hence, weight retention was a way of protecting one’s reputation. This situation was described in the ensuing statement by a South African focus group: “*In this community*, *people with HIV or AIDS are believed to sleep around and no-one wants people to say filthy things like that about them*.” [17].

#### Body image dissatisfaction

Due to both the social pressures to gain weight and the positive attributes associated with overweight, slender women experienced body image dissatisfaction and desired to gain more weight [16,29,32]. For example, a normal weight South African woman made the following statement: *"This is not my 'normal' weight*. *I would be happy and I will look more attractive*, *if I can gain more weight*.*"* [16]. A Kenyan woman also echoed a similar sentiment: “*I would like to be just a little bigger than this so I can feel more confident in myself*.” [32]. However, there were a few dissenting voices with some participants expressing a disinterest in being overweight or obese and others suggesting that the cultural acceptance of overweight and obesity was changing with increasing knowledge of the associated health risks [16,19].

#### Peer pressure among adolescent girls

Adolescent girls reported similar experiences as adult women: thinness signified HIV infection and influenced the willingness of obese girls to lose weight, and being voluptuous was regarded as more attractive [21,24,31]. A key difference, however, was that peer-related victimization was an important influence on the body size and shape ideals of adolescent girls. [18,21,24,31]. As described by one South African adolescent girl, those who did not fit into the preferred curvaceous ideal were shamed and labeled as HIV-infected persons: *"Sometimes you find that the other girls is [sic] having curves and you don't have it*. *If she saw you she is going to call you by all names [sic] saying that you don't have curves*, *you're thin as if you're HIV positive*. *you don't have buttocks*.*”* [21].

Unlike adult women, being overweight and obese was not socially accepted, and girls with such body sizes were victimized [18,24,31]. A quote by a Batswana adolescent girl depicted the issue: *"…usually when you around this from 6 to 10 [sic] when your body is this big you are more*, *as an adolescent you are more*, *you are going to be teased like you are likely to get your feelings hurt…*..*they are going to tease you because you have grown too fat*, *they isolate you*.*”* [31].

Further, muscularity in girls was linked with poverty and muscular girls were bullied [21]. An excerpt from the statements made by adolescent girls in a South African focus group discussion portrayed the situation: *"oh*, *they [the boys] laugh at them [the girls]*. *They told them that they have 'mapotirsi' [muscle]*, *and they have a hard skin because they work hard at home*.*”* [21].

However, there was evidence from a few of the studies that the opinions of family members also played a role in shaping adolescent body size preferences [24,31]. A South African adolescent girl in focus group discussions reported: "*My mother prefers a fat person to a slender person because she said when a person is slender it looks like that person is ill so because of that I ended up feeling proud for being fat*…” [24].

### Barriers to healthy food choices

Two sub-themes related to the aforementioned theme were identified for adult women. Adult females indicated that migration and high cost of healthy foods were reasons for unhealthy food choices. For adolescents, the affordability of healthy foods combined with a poor food environment at schools were barriers to healthy food choices. Similar to what was observed in the body shape and size ideal theme, peer pressure was also an important factor in adolescent food choices.

#### Migration

Migration was an important determinant of food choice among some adult women [20]. For Congolese and Zimbabwean migrant women in South Africa being away from home prevented them from making their traditional foods, which consisted of mostly plant-based meals, prompting them to compensate with fast foods. A Zimbabwean woman discussed the dietary transition she experienced in South Africa: "*There in Zimbabwe I used to eat our own traditional foods*. *[*. . . .*] Yes*, *but with this one it was totally different*. *[with this pregnancy] I wanted sweet stuff*, *fast foods*. *I almost ate fast food for this one I didn't cook for myself*. *I was lazy even here at home*. *I think it is because*. . . . *it is difficult for me to have our own traditional food*. *So I had to force myself to have an alternative*.*"* [20].

#### High cost of healthy foods

For other adult women and adolescent girls, the cost of food or poverty was a barrier to eating a more balanced diet [19,20,25–27]. A South African woman explained the barriers to healthy eating in her community: “*The cucumber and veggies and stuff are expensive*. *All the food with fat are cheap*, *vetkoek [deep-fried dough]*, *derms [sheep intestines]*, *pork*.” [19].

#### Peer pressure and lack of autonomy among adolescent girls

Adolescent girls experienced peer pressure to conform to the food choices of their friends [25,26,31]. A Batswana adolescent girl described her personal experience in the ensuing quote: "*For example when you are a group of friends and you are eating your healthy meal of like green salad and then everybody is having like pies*, *fizzy drinks*, *people will be looking like 'Wow*! *Wow*! *Eish*! *[an expression of surprise]*" [31]. Additionally, adolescents lacked autonomy in meals eaten at home, which were not always healthy [25].

#### Unhealthy food environment in schools

Compounding the problem for adolescents was that the food environment in secondary schools comprised of unhealthy foods [25–27,31]. A South African adolescent girl provided some specifics: *"During first school break we buy kotas [sandwich filled with chips*, *meat*, *cheese etc*.*] and cold drink*, *the next one crisps*, *the other one during study time we buy sweets and chocolate*.*"* [27]. Moreover, fruits were expensive, and in schools where free meals were provided by the school administration, they were reported to be unpleasant, further prompting the purchase of convenience foods [25–27]. Illustrative quotes from a South African adolescent girl are presented: *"I don't feel good about the free food we get at school*, *because they don't cook well*. *After eating it*, *I have stomach cramps*, *so we decided to stop eating the free food at school*. *If we don't have money for lunch*, *we just walk around the schoolyard until lunch is over; if we have some money we buy vetkoek and niknaks [cheese puffs] (from vendors)…"* [26].

### Barriers to engaging in physical activity

Five sub-themes were identified under the major theme of barriers to engaging in physical activity. The sub-theme of intrapersonal factors such as time and lack of knowledge was specific to adult women. Subthemes that were common to both adolescent girls and adult women were community safety, lack of recreational opportunities, and various forms of discrimination. Pregnant women comprising both adolescents and adult women reported several concerns including inadequate knowledge of appropriate physical activity during pregnancy. Further descriptions of these sub-themes with supporting quotes are presented below.

#### Intrapersonal factors

Time constraints, the potential for self-injury and lack of knowledge about appropriate physical activity were some of the personal concerns raised by adult women [23,30]. To exemplify this issue, a Ghanaian woman was reported as saying: "*It takes a lot of time in the day and we don't have that time… "I try to do exercises that are safe for me*, *for my age*, *but I don't always know what I can and cannot do…*.*"* [30]. Additionally, some of the interviewed women were not knowledgeable about the various forms of physical activity. For instance, when some adult women were asked about physical activities they engaged in, they often described activities of daily living [23,28,32]. This response to questions on the meanings of physical activity by a Kenyan woman exemplifies this finding: “*Someone who is active*, *works hard and is not lazy*. *An active person does things including working on the shamba (farm)*, *taking care of their families*, *cleaning their houses and keeps their yard looking good*.” [32].

#### Community safety

Adolescent girls, pregnant women and community informants regarded community safety as a barrier to engaging in physical activity [21,22,25]. This problem was expressed by a South African Male Youth Leader: “*There are people who are observing when we go to work or other places; when it is a woman and she is left alone*, *they are thinking of going there to rape her*.*”* [21]. A pregnant South African woman also expressed concern about neighborhood safety: “*Maybe if there was a way of doing exercise together it may be safe for us*. *It is not safe out there for walking*.*”* [22].

#### Lack of recreational opportunities

The paucity of recreational facilities or opportunities were common complaints among pregnant and non-pregnant adult women [19,22,23,28]. A South African pregnant woman described the situation: “*There are no facilities to exercise in my community…There is nothing other than sex*, *lots of it…”* [22]. School teachers also highlighted the problem of inadequate facilities when discussing barriers to physical activity among adolescent girls [21]. A male sports teacher was reported as saying: “*When they [girls] are busy playing [netball]*, *you find that a car of a teacher wants to go out and it disturbs them*. *Sometimes when they are busy playing you find that some of the students are coming out of the class and they will pass via the ground where they are playing*, *and they get disturbed*.*”* [21].

#### Discrimination

It was generally perceived that exercise was an activity for children and participation by adolescents and adult women was atypical [19,32]. These perceptions are illustrated by the ensuing quote from a South African focus group discussion: "*It's not important what they are doing because they look at the person and undermine them and think that this person is too old for the thing that he/she is doing*.” [19]. Further support is also provided by this excerpt from an in-depth interview of a Kenyan woman: “*I am now a mother and people would think I lost my mind if they saw me running and playing volleyball*. *This is for children and not for mothers like me*.*”* [32].

Age-based discrimination in physical activity was also reported to exist in schools [21]. South African adolescent girls in the 16 to 19-year age group revealed this issue in a focus group discussion: "*Even at school when you want to participate in athletics*, *they will tell you that you are over-age*, *so you become discouraged*.” [21]. Additionally, institutions and key stakeholders perpetuated gender inequalities in physical activity participation through inadequate maintenance and poor support for female sporting events [21]. A South African Male Sports Teacher explained in an in-depth interview: “*Our soccer pitch for boys is good*, *but for girls it's not good because the [netball] ground is inside the school yard and their place is too small*. *Mr*. *'M' is an owner of S [business] and he is sponsoring all the schools*. *He concentrates on the under-21s only*, *and he is doing it for boys only*. *Girls are suffering when it comes to competition and they don't have enough games*, *their games are very scarce*. . . .. . . . . .*To have sponsor [sic] for girls it is scarce*, *especially for netball or ladies’ soccer*. *Girls are left behind but for boys*, *everything is going well*.” [21]. Finally, overweight adolescent girls believed they were on the receiving end of weight bias from school officials who negatively perceived their sporting abilities and restricted them from participating in school events [18]. One South African adolescent girl spoke of her experience: “*Like me*, *I was always left out of sports teams in school because it was believed that fat people could not do well in sports*. *I spend [sic] most of my school years trying to prove myself*, *which has all been in vain*.*”*[18].

#### Pregnancy-related concerns

Pregnant women believed that physical activity could potentially harm them or their unborn child [22,28]. A South African woman explained: "*Maybe if you run a lot you can make the baby sore or if you change direction all of a sudden or you run into something you can hurt your baby*." [22]. They also reported receiving inadequate physical activity guidance from health care professionals, causing them to rely on family and friends for advice [22,28]. However, as illustrated in the following quote by a South African woman, pregnant women were often discouraged by friends and family from initiating any form of physical activity: “*My family…they used to just spoil me*, *but no*, *don’t do this*, *don’t go down*, *don’t pick it up*, *don’t do this*, *you know*? *So*, *you get spoiled easily*, *and it’s easy for you to go*, *ah*, *I don’t feel like doing this*, *and get so lazy…”*[28].

## Discussion and conclusion

To the best of our knowledge, this is the first qualitative research synthesis surfacing the contextual factors that predispose adult women and adolescent girls to overweight and obesity in sub-Saharan African countries. Our findings were grouped into three overarching themes consisting of body shape and size ideals, barriers to healthy eating, and barriers to physical activity. However, we found that cultural and social norms were connecting webs shaping attitudes, perceptions, and behaviors related to body size preferences, food choices and recreational physical activity for women and girls.

Cultural conceptions of ideal adult female bodies as large and/or voluptuous were consistent across studies in the four countries featured in this synthesis [16,29,31,32], and seemingly across both low and high socioeconomic status [29,31]. As illustrated by Eknoyan, positive connotations of female adiposity are not novel and were highly prevalent in the West up until the early 20^th^ century [33]. Eknoyan argued that such ideas resulted from the evolutionary advantage bestowed by excess fat in conditions of famine [33]. Therefore, since many SSA countries remain in low-income status or transitioned from low income to middle-income status in recent years [34], it is unsurprising that ideals of female body sizes and shape that promote adiposity still persist. However, these ideals may become detrimental as they come in contact with the increasing globalization of food systems coupled with the massive marketing of obesogenic foods, and the technological transition aiding the preponderance of sedentary lifestyles [35]. In other communities where large body size ideals have been observed, such as the African American community in the United States, the prevalence of obesity among women was greater than that of other racial groups [36–38]. Additionally, the presence of HIV stigma and the notion that thinness and weight loss were associated with HIV infection within SSA countries may exacerbate the situation: as we noted in the qualitative synthesis that overweight women were reluctant to lose weight to avoid stigma [17,19,21].

Among adolescents, however, we noted a lower social acceptance of overweight and obesity expressed through weight-related victimization involving bullying and teasing from their peers [21,24,31]. Weight-related victimization, also termed weight stigma, has also been observed in the United States [39,40]. In a study conducted among high school teens, the majority of respondents reported that overweight students were teased in a mean way, ignored, avoided and ostracized from social events [40]. The widespread societal belief that overweight and obese individuals are lazy, and that weight stigma is a beneficial incentive for weight loss, may explain the victimization experiences associated with adiposity [41–43]. But the notion that weight stigma is beneficial lies in contrast to the research literature that shows that it precipitates deleterious effects such as binge eating, social isolation, decreased physical activity, and excessive weight gain [44,45].

It is also possible the influx of western ideals may further account for adolescents’ negative perception of overweight and obesity. Swami calls it the “globalization of the thin ideal” [46]. But given that thinness is typically associated with illness or HIV infection in South Africa and Botswana, the two countries from which studies of adolescents were included in this synthesis, western body size ideals probably lies in tension with cultural ideals leading to a somewhat “middle ground” in adolescent body size and shape ideals.

Addressing the cultural factors and social pressures linked to overweight requires the design and adoption of culturally sensitive interventions; one strategy for such intervention is the Person, Extended Family and Neighborhood Factors– 3 (PEN-3) model. The PEN-3 model is a theoretical framework used to foreground culture in the development, implementation and evaluation of health interventions [47]. For instance, in a depression prevention intervention for urban African American and Latino adolescents, the PEN-3 model guided the identification of individual, interpersonal and community level barriers and promoters of depression and this information was applied to improve the visual and linguistic appeal of the program publications [48]. With regards to our study’s findings, the PEN-3 model could be applied to develop interventions that address cultural and social conceptions of ideal body sizes, to deconstruct myths surrounding the link between weight loss and HIV, as well as promote an environment where all community members are accepted irrespective of HIV and weight status. Peer bullying interventions that tackle body shaming and weight-related victimization in adolescents are also warranted. As extant interventions show a small to medium effect on weight-biased attitudes and beliefs, novel interventions informed by the findings of this review are needed [48].

Our research synthesis also revealed a nutrition transition from traditional to convenience foods. Key factors shaping food choices included migration, cost, peer pressure, and the food environment [19,20,26,27,31]. Women and girls also encountered barriers to physical activity including ageism, gender-based discrimination in funding for female sporting events, community safety concerns, and a paucity of facilities that support women’s engagement in physical activity [21,22,25,32]. Addressing the barriers to healthy food choices and engagement in physical activity identified in this research synthesis requires the development of national guidelines and the implementation of recommended evidence-based policy and program options from the WHO Global Action Plan [5], which have been endorsed by Member States including SSA countries. One recommended strategy to improve the food environment within institutions and in the broader environment from the Global Action Plan are policy measures that engage food producers and processors to increase the affordability and availability of fruits and vegetables; and substitute saturated fats in processed foods with unsaturated fats [5]. The Food and Agriculture Organization also stresses action to improve information access on the nutritional content and health consequences of different foods to enhance better decision making in food purchases [49]. Regarding physical activity, policy actions to promote female participation and reduce the gender discrimination issues identified in this synthesis must be informed by the Sustainable Development Goal Target 5.9, which is to “adopt and strengthen sound policies and enforceable legislation for the promotion of gender equality and the empowerment of all women and girls at all levels” [8]. Addressing overweight and obesity poses a challenge for even advanced economies, with no reversals in the trajectory recorded in any country [50]. Nonetheless, given that several SSA countries are still in the nascent stages of the epidemic, an opportunity exists to tackle the problem before it spirals out of control.

To do this effectively requires collaboration with community members. Community-based participatory research methods provide opportunities for community members to participate in the design of interventions and have been found to be effective in the prevention and control of obesity [36,47]. With respect to our study’s findings on the poor food environment found in schools, suggested measures include consulting adolescents and their parents to identify nutritious foods that are acceptable and culturally appropriate. Additionally, adolescents’ acceptance and interest in the consumption of healthy meals could be enhanced through the development of school-led community gardens. Moreover, incentives could be offered to local farmers to support them in the production and supply of fruits and vegetables to local schools. Collectively, these strategies could improve the food environment, adolescents’ food choices and decrease peer pressure associated with choosing convenience food.

The findings of this synthesis should be viewed in the light of the following limitations. First, we generalize findings from studies conducted in different countries with diverse qualitative methods and samples. While we identified common themes across studies, the extent to which these themes apply within individual countries is unknown. Another key limitation of this synthesis is that only four SSA countries were examined, which hampers the transferability of the findings to countries outside of those included in this study. It is therefore advised that caution should be exercised in the applications of the findings to a different SSA country. Additionally, within included studies, the categorization of socioeconomic status by investigators was unclear, and explicit definition of ideal body sizes in quantitative terms, such as BMI, was unavailable. However, the insights gained from this synthesis provide a background on potential contributing factors related to female overweight and obesity in sub-Saharan Africa to guide the design of additional studies that assess the contribution of these factors to overweight and obesity in order to prioritize intervention targets. This study indicates that there is a huge gap in qualitative evidence on factors related to overweight and obesity, food choices and physical activity across African countries.

In conclusion, this research synthesis of qualitative studies identified contextual factors that predispose adolescent girls and adult women to overweight and obesity in SSA countries. Body size and shape ideals, food choices and physical activity participation were influenced by several determinants including sociocultural factors, migration, ageism, institutional factors, self-efficacy and peer pressure. We also found a number of adverse effects from the institutionalization of body size and shape ideals including weight-related victimization among adolescents and stigmatization of thinness due to a perceived link with HIV. Community-based participatory research, policy design and implementation and multi-sectoral efforts to prevent and control female overweight and obesity in SSA communities are warranted.

## Supporting information

S1 File Supplementary appendix. (DOCX)

S2 File Critical appraisal forms. (ZIP)
