# Supplementary material for: A qualitative research synthesis of contextual factors contributing to female overweight and obesity over the life course in sub-Saharan Africa
Source: PLoS One. 2019 Nov 4;14(11):e0224612. doi: 10.1371/journal.pone.0224612 (PMC6827897; doi:10.1371/journal.pone.0224612)
Supplement: S2 File — (ZIP) [file pone.0224612.s002.zip › Critical Appraisal/Critical Appraisal documents.pdf]

Brown - University of pennsylvania — aim 4

CASP

Critical Appraisal Skills Programme

www.casp-uk.net
info@casp-uk.net
Summertown Pavilion, Middle Way Oxford OX2 7LG

Topic: Adolescent and adult perceptions of adolescent diets, physical activity, Body Size, and obesity prevention in Botswana.

- Christian Nwabueze.

CASP Checklist: 10 questions to help you make sense of a **Qualitative** research

How to use this appraisal tool: Three broad issues need to be considered when appraising a qualitative study:

- Are the results of the study valid? (Section A)
- What are the results? (Section B)
- Will the results help locally? (Section C)

The 10 questions on the following pages are designed to help you think about these issues systematically. The first two questions are screening questions and can be answered quickly. If the answer to both is "yes", it is worth proceeding with the remaining questions. There is some degree of overlap between the questions, you are asked to record a "yes", "no" or "can't tell" to most of the questions. A number of italicised prompts are given after each question. These are designed to remind you why the question is important. Record your reasons for your answers in the spaces provided.

About: These checklists were designed to be used as educational pedagogic tools, as part of a workshop setting, therefore we do not suggest a scoring system. The core CASP checklists (randomised controlled trial & systematic review) were based on JAMA 'Users' guides to the medical literature 1994 (adapted from Guyatt GH, Sackett DL, and Cook DJ), and piloted with health care practitioners.

For each new checklist, a group of experts were assembled to develop and pilot the checklist and the workshop format with which it would be used. Over the years overall adjustments have been made to the format, but a recent survey of checklist users reiterated that the basic format continues to be useful and appropriate.

Referencing: we recommend using the Harvard style citation, i.e.: *Critical Appraisal Skills Programme (2018). CASP (insert name of checklist i.e. Qualitative) Checklist. [online] Available at: URL. Accessed: Date Accessed.*

©CASP this work is licensed under the Creative Commons Attribution – Non-Commercial-Share A like. To view a copy of this license, visit http://creativecommons.org/licenses/by-nc-sa/3.0/ www.casp-uk.net

CASP
Critical Appraisal Skills Programme

Paper for appraisal and reference: ....

Section A: Are the results valid?

1. Was there a clear statement of the aims of the research?			HINT: Consider
	Yes	✓	• what was the goal of the research
	Can't Tell		• why it was thought important
	No		• its relevance

Comments:

2. Is a qualitative methodology appropriate?			HINT: Consider
	Yes	✓	• If the research seeks to interpret or illuminate the actions and/or subjective experiences of research participants
	Can't Tell		• Is qualitative research the right methodology for addressing the research goal
	No		

Comments:

Is it worth continuing?

3. Was the research design appropriate to address the aims of the research?			HINT: Consider
	Yes	✓	• if the researcher has justified the research design (e.g. have they discussed how they decided which method to use)
	Can't Tell		
	No		

Comments:

CASP
Critical Appraisal Skills Programme

4. Was the recruitment strategy appropriate to the aims of the research?

Yes	✓
Can't Tell	
No	

HINT: Consider
- If the researcher has explained how the participants were selected
- If they explained why the participants they selected were the most appropriate to provide access to the type of knowledge sought by the study
- If there are any discussions around recruitment (e.g. why some people chose not to take part)

Comments:

5. Was the data collected in a way that addressed the research issue?

Yes	✓
Can't Tell	
No	

HINT: Consider
- If the setting for the data collection was justified
- If it is clear how data were collected (e.g. focus group, semi-structured interview etc.)
- If the researcher has justified the methods chosen
- If the researcher has made the methods explicit (e.g. for interview method, is there an indication of how interviews are conducted, or did they use a topic guide)
- If methods were modified during the study. If so, has the researcher explained how and why
- If the form of data is clear (e.g. tape recordings, video material, notes etc.)
- If the researcher has discussed saturation of data

Comments:

CASP
Critical Appraisal Skills Programme

6. Has the relationship between researcher and participants been adequately considered?

Yes	
Can't Tell	
No	✓

HINT: Consider
- If the researcher critically examined their own role, potential bias and influence during (a) formulation of the research questions (b) data collection, including sample recruitment and choice of location
- How the researcher responded to events during the study and whether they considered the implications of any changes in the research design

Comments:

Section B: What are the results?

7. Have ethical issues been taken into consideration?

Yes	✓
Can't Tell	
No	

HINT: Consider
- If there are sufficient details of how the research was explained to participants for the reader to assess whether ethical standards were maintained
- If the researcher has discussed issues raised by the study (e.g. issues around informed consent or confidentiality or how they have handled the effects of the study on the participants during and after the study)
- If approval has been sought from the ethics committee

Comments:

CASP
Critical Appraisal Skills Programme

8. Was the data analysis sufficiently rigorous?		HINT: Consider
	Yes	✓
	Can't Tell	
	No	

HINT: Consider
- If there is an in-depth description of the analysis process
- If thematic analysis is used. If so, is it clear how the categories/themes were derived from the data
- Whether the researcher explains how the data presented were selected from the original sample to demonstrate the analysis process
- If sufficient data are presented to support the findings
- To what extent contradictory data are taken into account
- Whether the researcher critically examined their own role, potential bias and influence during analysis and selection of data for presentation

Comments:

9. Is there a clear statement of findings?		
	Yes	✓
	Can't Tell	
	No	

HINT: Consider whether
- If the findings are explicit
- If there is adequate discussion of the evidence both for and against the researcher's arguments
- If the researcher has discussed the credibility of their findings (e.g. triangulation, respondent validation, more than one analyst)
- If the findings are discussed in relation to the original research question

Comments:

CASP
Critical Appraisal
Skills Programme

Section C: Will the results help locally?

10. How valuable is the research?

HINT: Consider
- If the researcher discusses the contribution the study makes to existing knowledge or understanding (e.g. do they consider the findings in relation to current practice or policy, or relevant research-based literature
- If they identify new areas where research is necessary
- If the researchers have discussed whether or how the findings can be transferred to other populations or considered other ways the research may be used

Comments:

Perceptions about body image and sizes among Black African girls living in Cape Town

IFEOMA 0201DIEGWU

7/10

CASP

Critical Appraisal Skills Programme

www.casp-uk.net

info@casp-uk.net

Summertown Pavilion, Middle Way Oxford OX2 7LG

CASP Checklist: 10 questions to help you make sense of a **Qualitative** research

How to use this appraisal tool: Three broad issues need to be considered when appraising a qualitative study:

- Are the results of the study valid? (Section A)
- What are the results? (Section B)
- Will the results help locally? (Section C)

The 10 questions on the following pages are designed to help you think about these issues systematically. The first two questions are screening questions and can be answered quickly. If the answer to both is "yes", it is worth proceeding with the remaining questions. There is some degree of overlap between the questions, you are asked to record a "yes", "no" or "can't tell" to most of the questions. A number of italicised prompts are given after each question. These are designed to remind you why the question is important. Record your reasons for your answers in the spaces provided.

About: These checklists were designed to be used as educational pedagogic tools, as part of a workshop setting, therefore we do not suggest a scoring system. The core CASP checklists (randomised controlled trial & systematic review) were based on JAMA 'Users' guides to the medical literature 1994 (adapted from Guyatt GH, Sackett DL, and Cook DJ), and piloted with health care practitioners.

For each new checklist, a group of experts were assembled to develop and pilot the checklist and the workshop format with which it would be used. Over the years overall adjustments have been made to the format, but a recent survey of checklist users reiterated that the basic format continues to be useful and appropriate.

Referencing: we recommend using the Harvard style citation, i.e.: *Critical Appraisal Skills Programme (2018). CASP (insert name of checklist i.e. Qualitative) Checklist. [online] Available at: URL. Accessed: Date Accessed.*

1433

©CASP this work is licensed under the Creative Commons Attribution – Non-Commercial-Share A like. To view a copy of this license, visit http://creativecommons.org/licenses/by-nc-sa/3.0/ www.casp-uk.net

Perceptions about body image and sizes among black african girls living in cape town. Puoane et al 2010

Factors influencing weight control/practices amongst adolescent girls in Vhembe District of Limpopo Province, SA

CASP
Critical Appraisal Skills Programme

Paper for appraisal and reference:

Section A: Are the results valid?

(1)

1. Was there a clear statement of the aims of the research?

Yes [✓] Can't Tell [ ] No [ ]

HINT: Consider
- what was the goal of the research
- why it was thought important
- its relevance

Comments: The aim of the study was to examine the perception of black school girls aged 10 - [illegible] years about body size and body image. Knowledge of the age at which girls associate weight status with ~~attractives could~~ influence ~~interceptions~~.

2. Is a qualitative methodology appropriate?

Yes [✓] Can't Tell [ ] No [ ]

HINT: Consider
- If the research seeks to interpret or illuminate the actions and/or subjective experiences of research participants
- Is qualitative research the right methodology for addressing the research goal

Comments: A focus group discussions is a good forum to illuminate individual perspective / knowledge.

Is it worth continuing?

3. Was the research design appropriate to address the aims of the research?

Yes [ ] Can't Tell [ ] No [✓]

HINT: Consider
- if the researcher has justified the research design (e.g. have they discussed how they decided which method to use)

Comments: The choice of research design method was not discussed.

4. Was the recruitment strategy appropriate to the aims of the research?

Yes	✓
Can't Tell	
No	

HINT: Consider
- If the researcher has explained how the participants were selected
- If they explained why the participants they selected were the most appropriate to provide access to the type of knowledge sought by the study
- If there are any discussions around recruitment (e.g. why some people chose not to take part)

Comments:
The girls in this study came from a disadvantaged background. While this provides insight into the world view of people from such background, the generalisability is not clear. However, why only 60 girls were selected for the recruitment is questionable.

5. Was the data collected in a way that addressed the research issue?

Yes	✓
Can't Tell	
No	

HINT: Consider
- If the setting for the data collection was justified
- If it is clear how data were collected (e.g. focus group, semi-structured interview etc.)
- If the researcher has justified the methods chosen
- If the researcher has made the methods explicit (e.g. for interview method, is there an indication of how interviews are conducted, or did they use a topic guide)
- If methods were modified during the study. If so, has the researcher explained how and why
- If the form of data is clear (e.g. tape recordings, video material, notes etc.)
- If the researcher has discussed saturation of data

Comments:
Data collection process was clear. Data collection was done by tape recording in a focus group format. However, the setting of data collection is not clear.

6. Has the relationship between researcher and participants been adequately considered?

Yes	
Can't Tell	
No	✓

HINT: Consider

- If the researcher critically examined their own role, potential bias and influence during (a) formulation of the research questions (b) data collection, including sample recruitment and choice of location
- How the researcher responded to events during the study and whether they considered the implications of any changes in the research design

Comments:

No information on this

Section B: What are the results?

7. Have ethical issues been taken into consideration?

Yes	✓
Can't Tell	
No	

HINT: Consider

- If there are sufficient details of how the research was explained to participants for the reader to assess whether ethical standards were maintained
- If the researcher has discussed issues raised by the study (e.g. issues around informed consent or confidentiality or how they have handled the effects of the study on the participants during and after the study)
- If approval has been sought from the ethics committee

Comments:

Yes, ethical approval was received.

CASP
Critical Appraisal Skills Programme

8. Was the data analysis sufficiently rigorous?

Yes	
Can't Tell	✓
No	

HINT: Consider
- If there is an in-depth description of the analysis process
- If thematic analysis is used. If so, is it clear how the categories/themes were derived from the data
- Whether the researcher explains how the data presented were selected from the original sample to demonstrate the analysis process
- If sufficient data are presented to support the findings
- To what extent contradictory data are taken into account
- Whether the researcher critically examined their own role, potential bias and influence during analysis and selection of data for presentation *

Comments:
While it appears that the data analysis was rigorous, the researcher does not present any information on their reflexivity

9. Is there a clear statement of findings?

Yes	✓
Can't Tell	
No	

HINT: Consider whether
- If the findings are explicit
- If there is adequate discussion of the evidence both for and against the researcher's arguments
- If the researcher has discussed the credibility of their findings (e.g. triangulation, respondent validation, more than one analyst)
- If the findings are discussed in relation to the original research question

Comments:
A clear statement of findings was made, validation included more than one analyst but no attempt was made to validate the findings with respondents.

CASP
Critical Appraisal
Skills Programme

Section C: Will the results help locally?

10. How valuable is the research?

Yes

HINT: Consider
- If the researcher discusses the contribution the study makes to existing knowledge or understanding (e.g. do they consider the findings in relation to current practice or policy, or relevant research-based literature
- If they identify new areas where research is necessary
- If the researchers have discussed whether or how the findings can be transferred to other populations or considered other ways the research may be used

Comments:
The info on the importance of the research was shared in the discussion.

It seems that in a socially-disadvantaged community, there are varying opinions and perceptions on the advantages and disadvantages of thinness and fatness.

Thin girls have a negative perception of fatness ~~fat girls have a nega~~ ~~sinners~~ girls of all sizes have a somewhat negative perception of thinness.

Obese girls seem to have positive perception of fatness and obesity and thinness have the propensity to be stigmatised.

~~Parent~~ Parental ~~peep~~ attitudes towards thinness or fatness appear to be important

DONE

Tuesday: 8–5pm
Monday: 4–10
Friday: 4–9

CASP

Critical Appraisal Skills Programme

www.casp-uk.net
info@casp-uk.net
Summertown Pavilion, Middle Way Oxford OX2 7LG

Christian Nwabueze: Perceptions about body image and sizes among black African girls living in Cape Town.

CASP Checklist: 10 questions to help you make sense of a **Qualitative** research

How to use this appraisal tool: Three broad issues need to be considered when appraising a qualitative study:

- Are the results of the study valid? (Section A)
- What are the results? (Section B)
- Will the results help locally? (Section C)

The 10 questions on the following pages are designed to help you think about these issues systematically. The first two questions are screening questions and can be answered quickly. If the answer to both is "yes", it is worth proceeding with the remaining questions. There is some degree of overlap between the questions, you are asked to record a "yes", "no" or "can't tell" to most of the questions. A number of italicised prompts are given after each question. These are designed to remind you why the question is important. Record your reasons for your answers in the spaces provided.

About: These checklists were designed to be used as educational pedagogic tools, as part of a workshop setting, therefore we do not suggest a scoring system. The core CASP checklists (randomised controlled trial & systematic review) were based on JAMA 'Users' guides to the medical literature 1994 (adapted from Guyatt GH, Sackett DL, and Cook DJ), and piloted with health care practitioners.

For each new checklist, a group of experts were assembled to develop and pilot the checklist and the workshop format with which it would be used. Over the years overall adjustments have been made to the format, but a recent survey of checklist users reiterated that the basic format continues to be useful and appropriate.

Referencing: we recommend using the Harvard style citation, i.e.: *Critical Appraisal Skills Programme (2018). CASP (insert name of checklist i.e. Qualitative) Checklist. [online] Available at: URL. Accessed: Date Accessed.*

©CASP this work is licensed under the Creative Commons Attribution – Non-Commercial-Share A like. To view a copy of this license, visit http://creativecommons.org/licenses/by-nc-sa/3.0/ www.casp-uk.net

CASP
Critical Appraisal
Skills Programme

Paper for appraisal and reference:

Section A: Are the results valid?

1. Was there a clear statement of the aims of the research?

Yes ✓
Can't Tell
No

HINT: Consider
- what was the goal of the research
- why it was thought important
- its relevance

Comments:

2. Is a qualitative methodology appropriate?

Yes ✓
Can't Tell
No

HINT: Consider
- If the research seeks to interpret or illuminate the actions and/or subjective experiences of research participants
- Is qualitative research the right methodology for addressing the research goal

Comments:

Is it worth continuing?

3. Was the research design appropriate to address the aims of the research?

Yes ✓
Can't Tell
No

HINT: Consider
- if the researcher has justified the research design (e.g. have they discussed how they decided which method to use)

Comments:

4. Was the recruitment strategy appropriate to the aims of the research?

Yes	✓
Can't Tell	
No	

HINT: Consider
- If the researcher has explained how the participants were selected
- If they explained why the participants they selected were the most appropriate to provide access to the type of knowledge sought by the study
- If there are any discussions around recruitment (e.g. why some people chose not to take part)

Comments:

5. Was the data collected in a way that addressed the research issue?

Yes	✓
Can't Tell	
No	

HINT: Consider
- If the setting for the data collection was justified
- If it is clear how data were collected (e.g. focus group, semi-structured interview etc.)
- If the researcher has justified the methods chosen
- If the researcher has made the methods explicit (e.g. for interview method, is there an indication of how interviews are conducted, or did they use a topic guide)
- If methods were modified during the study. If so, has the researcher explained how and why
- If the form of data is clear (e.g. tape recordings, video material, notes etc.)
- If the researcher has discussed saturation of data

Comments:

CASP
Critical Appraisal Skills Programme

6. Has the relationship between researcher and participants been adequately considered?	Yes		HINT: Consider<br>• If the researcher critically examined their own role, potential bias and influence during (a) formulation of the research questions (b) data collection, including sample recruitment and choice of location<br>• How the researcher responded to events during the study and whether they considered the implications of any changes in the research design
	Can't Tell		
	No	✓	

Comments:

Section B: What are the results?

7. Have ethical issues been taken into consideration?	Yes	✓	HINT: Consider<br>• If there are sufficient details of how the research was explained to participants for the reader to assess whether ethical standards were maintained<br>• If the researcher has discussed issues raised by the study (e.g. issues around informed consent or confidentiality or how they have handled the effects of the study on the participants during and after the study)<br>• If approval has been sought from the ethics committee
	Can't Tell		
	No		

Comments:

8. Was the data analysis sufficiently rigorous?

Yes	✓
Can't Tell	
No	

HINT: Consider
- If there is an in-depth description of the analysis process
- If thematic analysis is used. If so, is it clear how the categories/themes were derived from the data
- Whether the researcher explains how the data presented were selected from the original sample to demonstrate the analysis process
- If sufficient data are presented to support the findings
- To what extent contradictory data are taken into account
- Whether the researcher critically examined their own role, potential bias and influence during analysis and selection of data for presentation

Comments:

9. Is there a clear statement of findings?

Yes	✓
Can't Tell	
No	

HINT: Consider whether
- If the findings are explicit
- If there is adequate discussion of the evidence both for and against the researcher's arguments
- If the researcher has discussed the credibility of their findings (e.g. triangulation, respondent validation, more than one analyst)
- If the findings are discussed in relation to the original research question

Comments:

CASP
Critical Appraisal Skills Programme

Section C: Will the results help locally?

10. How valuable is the research?

Yes ✓

Can't tell

No

HINT: Consider

- If the researcher discusses the contribution the study makes to existing knowledge or understanding (e.g. do they consider the findings in relation to current practice or policy, or relevant research-based literature
- If they identify new areas where research is necessary
- If the researchers have discussed whether or how the findings can be transferred to other populations or considered other ways the research may be used

Comments:

Obesity Among Women in Rural Kenya: Knowledge, Beliefs & Perceptions
IFEOMA OZODIEGWU

CASP
Critical Appraisal Skills Programme

9/10

www.casp-uk.net
info@casp-uk.net
Summertown Pavilion, Middle Way Oxford OX2 7LG

CASP Checklist: 10 questions to help you make sense of a **Qualitative** research

How to use this appraisal tool: Three broad issues need to be considered when appraising a qualitative study:

- Are the results of the study valid? (Section A)
- What are the results? (Section B)
- Will the results help locally? (Section C)

The 10 questions on the following pages are designed to help you think about these issues systematically. The first two questions are screening questions and can be answered quickly. If the answer to both is "yes", it is worth proceeding with the remaining questions. There is some degree of overlap between the questions, you are asked to record a "yes", "no" or "can't tell" to most of the questions. A number of italicised prompts are given after each question. These are designed to remind you why the question is important. Record your reasons for your answers in the spaces provided.

About: These checklists were designed to be used as educational pedagogic tools, as part of a workshop setting, therefore we do not suggest a scoring system. The core CASP checklists (randomised controlled trial & systematic review) were based on JAMA 'Users' guides to the medical literature 1994 (adapted from Guyatt GH, Sackett DL, and Cook DJ), and piloted with health care practitioners.

For each new checklist, a group of experts were assembled to develop and pilot the checklist and the workshop format with which it would be used. Over the years overall adjustments have been made to the format, but a recent survey of checklist users reiterated that the basic format continues to be useful and appropriate.

Referencing: we recommend using the Harvard style citation, i.e.: *Critical Appraisal Skills Programme (2018). CASP (insert name of checklist i.e. Qualitative) Checklist. [online] Available at: URL. Accessed: Date Accessed.*

©CASP this work is licensed under the Creative Commons Attribution – Non-Commercial-Share A like. To view a copy of this license, visit http://creativecommons.org/licenses/by-nc-sa/3.0/ www.casp-uk.net

CASP
Critical Appraisal
Skills Programme

Obesity among women in rural Kenya: Knowledges beliefs & perceptions.

N1

Paper for appraisal and reference:

Section A: Are the results valid?

1. Was there a clear statement of the aims of the research?

Yes ✓ | Can't Tell | No

HINT: Consider
- what was the goal of the research
- why it was thought important
- its relevance

Comments:

2. Is a qualitative methodology appropriate?

Yes ✓ | Can't Tell | No

HINT: Consider
- If the research seeks to interpret or illuminate the actions and/or subjective experiences of research participants
- Is qualitative research the right methodology for addressing the research goal

Comments:

Is it worth continuing?

3. Was the research design appropriate to address the aims of the research?

Yes ✓ | Can't Tell | No

HINT: Consider
- if the researcher has justified the research design (e.g. have they discussed how they decided which method to use)

Comments:

4. Was the recruitment strategy appropriate to the aims of the research?

Yes	✓
Can't Tell	
No	

HINT: Consider
- If the researcher has explained how the participants were selected
- If they explained why the participants they selected were the most appropriate to provide access to the type of knowledge sought by the study
- If there are any discussions around recruitment (e.g. why some people chose not to take part)

Comments:

5. Was the data collected in a way that addressed the research issue?

Yes	✓ (scribbled out)
Can't Tell	
No	✓

HINT: Consider
- If the setting for the data collection was justified
- If it is clear how data were collected (e.g. focus group, semi-structured interview etc.)
- If the researcher has justified the methods chosen
- If the researcher has made the methods explicit (e.g. for interview method, is there an indication of how interviews are conducted, or did they use a topic guide)
- If methods were modified during the study. If so, has the researcher explained how and why
- If the form of data is clear (e.g. tape recordings, video material, notes etc.)
- If the researcher has discussed saturation of data

Comments:

CASP
Critical Appraisal
Skills Programme

6. Has the relationship between researcher and participants been adequately considered?

Yes ✓

Can't Tell

No

HINT: Consider

- If the researcher critically examined their own role, potential bias and influence during (a) formulation of the research questions (b) data collection, including sample recruitment and choice of location
- How the researcher responded to events during the study and whether they considered the implications of any changes in the research design

Comments:

Section B: What are the results?

7. Have ethical issues been taken into consideration?

Yes ✓

Can't Tell

No

HINT: Consider

- If there are sufficient details of how the research was explained to participants for the reader to assess whether ethical standards were maintained
- If the researcher has discussed issues raised by the study (e.g. issues around informed consent or confidentiality or how they have handled the effects of the study on the participants during and after the study)
- If approval has been sought from the ethics committee

Comments:

CASP
Critical Appraisal Skills Programme

8. Was the data analysis sufficiently rigorous?

Yes	✓
Can't Tell	
No	

HINT: Consider
- If there is an in-depth description of the analysis process
- If thematic analysis is used. If so, is it clear how the categories/themes were derived from the data
- Whether the researcher explains how the data presented were selected from the original sample to demonstrate the analysis process
- If sufficient data are presented to support the findings
- To what extent contradictory data are taken into account
- Whether the researcher critically examined their own role, potential bias and influence during analysis and selection of data for presentation

Comments:

9. Is there a clear statement of findings?

Yes	✓
Can't Tell	
No	

HINT: Consider whether
- If the findings are explicit
- If there is adequate discussion of the evidence both for and against the researcher's arguments
- If the researcher has discussed the credibility of their findings (e.g. triangulation, respondent validation, more than one analyst)
- If the findings are discussed in relation to the original research question

Comments:

CASP
Critical Appraisal Skills Programme

Section C: Will the results help locally?

10. How valuable is the research?

Yes

HINT: Consider
- If the researcher discusses the contribution the study makes to existing knowledge or understanding (e.g. do they consider the findings in relation to current practice or policy, or relevant research-based literature
- If they identify new areas where research is necessary
- If the researchers have discussed whether or how the findings can be transferred to other populations or considered other ways the research may be used

Comments:

Ann Mugo → Obesity among Women in Rural Kenya: Knowledge, Beliefs, and Perceptions.

↳ Christian Nwabueze.

CASP
Critical Appraisal
Skills Programme

www.casp-uk.net

info@casp-uk.net

Summertown Pavilion, Middle
Way Oxford OX2 7LG

CASP Checklist: 10 questions to help you make sense of a **Qualitative** research

How to use this appraisal tool: Three broad issues need to be considered when appraising a qualitative study:

- Are the results of the study valid? (Section A)
- What are the results? (Section B)
- Will the results help locally? (Section C)

The 10 questions on the following pages are designed to help you think about these issues systematically. The first two questions are screening questions and can be answered quickly. If the answer to both is "yes", it is worth proceeding with the remaining questions. There is some degree of overlap between the questions, you are asked to record a "yes", "no" or "can't tell" to most of the questions. A number of italicised prompts are given after each question. These are designed to remind you why the question is important. Record your reasons for your answers in the spaces provided.

About: These checklists were designed to be used as educational pedagogic tools, as part of a workshop setting, therefore we do not suggest a scoring system. The core CASP checklists (randomised controlled trial & systematic review) were based on JAMA 'Users' guides to the medical literature 1994 (adapted from Guyatt GH, Sackett DL, and Cook DJ), and piloted with health care practitioners.

For each new checklist, a group of experts were assembled to develop and pilot the checklist and the workshop format with which it would be used. Over the years overall adjustments have been made to the format, but a recent survey of checklist users reiterated that the basic format continues to be useful and appropriate.

Referencing: we recommend using the Harvard style citation, i.e.: *Critical Appraisal Skills Programme (2018). CASP (insert name of checklist i.e. Qualitative) Checklist. [online] Available at: URL. Accessed: Date Accessed.*

©CASP this work is licensed under the Creative Commons Attribution – Non-Commercial-Share A like. To view a copy of this license, visit http://creativecommons.org/licenses/by-nc-sa/3.0/ www.casp-uk.net

CASP

Critical Appraisal Skills Programme

Paper for appraisal and reference: ....

Section A: Are the results valid?

1. Was there a clear statement of the aims of the research?

Yes	✓
Can't Tell	
No	

HINT: Consider
- what was the goal of the research
- why it was thought important
- its relevance

Comments:

2. Is a qualitative methodology appropriate?

Yes	✓
Can't Tell	
No	

HINT: Consider
- If the research seeks to interpret or illuminate the actions and/or subjective experiences of research participants
- Is qualitative research the right methodology for addressing the research goal

Comments:

Is it worth continuing?

3. Was the research design appropriate to address the aims of the research?

Yes	✓
Can't Tell	
No	

HINT: Consider
- if the researcher has justified the research design (e.g. have they discussed how they decided which method to use)

Comments:

4. Was the recruitment strategy appropriate to the aims of the research?

Yes ☑
Can't Tell ☐
No ☐

HINT: Consider
- If the researcher has explained how the participants were selected
- If they explained why the participants they selected were the most appropriate to provide access to the type of knowledge sought by the study
- If there are any discussions around recruitment (e.g. why some people chose not to take part)

Comments:

5. Was the data collected in a way that addressed the research issue?

Yes ☑
Can't Tell ☐
No ☐

HINT: Consider
- If the setting for the data collection was justified
- If it is clear how data were collected (e.g. focus group, semi-structured interview etc.)
- If the researcher has justified the methods chosen
- If the researcher has made the methods explicit (e.g. for interview method, is there an indication of how interviews are conducted, or did they use a topic guide)
- If methods were modified during the study. If so, has the researcher explained how and why
- If the form of data is clear (e.g. tape recordings, video material, notes etc.)
- If the researcher has discussed saturation of data

Comments:

6. Has the relationship between researcher and participants been adequately considered?

Yes	✓
Can't Tell	
No	

HINT: Consider
- If the researcher critically examined their own role, potential bias and influence during (a) formulation of the research questions (b) data collection, including sample recruitment and choice of location
- How the researcher responded to events during the study and whether they considered the implications of any changes in the research design

Comments:

Section B: What are the results?

7. Have ethical issues been taken into consideration?

Yes	✓
Can't Tell	
No	

HINT: Consider
- If there are sufficient details of how the research was explained to participants for the reader to assess whether ethical standards were maintained
- If the researcher has discussed issues raised by the study (e.g. issues around informed consent or confidentiality or how they have handled the effects of the study on the participants during and after the study)
- If approval has been sought from the ethics committee

Comments:

CASP
Critical Appraisal
Skills Programme

8. Was the data analysis sufficiently rigorous?

Yes	✓
Can't Tell	
No	

HINT: Consider
- If there is an in-depth description of the analysis process
- If thematic analysis is used. If so, is it clear how the categories/themes were derived from the data
- Whether the researcher explains how the data presented were selected from the original sample to demonstrate the analysis process
- If sufficient data are presented to support the findings
- To what extent contradictory data are taken into account
- Whether the researcher critically examined their own role, potential bias and influence during analysis and selection of data for presentation

Comments:

9. Is there a clear statement of findings?

Yes	✓
Can't Tell	
No	

HINT: Consider whether
- If the findings are explicit
- If there is adequate discussion of the evidence both for and against the researcher's arguments
- If the researcher has discussed the credibility of their findings (e.g. triangulation, respondent validation, more than one analyst)
- If the findings are discussed in relation to the original research question

Comments:

CASP
Critical Appraisal
Skills Programme

Section C: Will the results help locally?

10. How valuable is the research?

HINT: Consider
- If the researcher discusses the contribution the study makes to existing knowledge or understanding (e.g. do they consider the findings in relation to current practice or policy, or relevant research-based literature
- If they identify new areas where research is necessary
- If the researchers have discussed whether or how the findings can be transferred to other populations or considered other ways the research may be used

Comments:

IFEOMA OZODIEGWU: Perceived ideal body size of Ghanaian women – "Not too skinny but not fat"

CASP

Critical Appraisal Skills Programme

7/10

www.casp-uk.net

info@casp-uk.net

Summertown Pavilion, Middle Way Oxford OX2 7LG

IFEOMA OZODIEGWU.

CASP Checklist: 10 questions to help you make sense of a **Qualitative** research

How to use this appraisal tool: Three broad issues need to be considered when appraising a qualitative study:

- Are the results of the study valid? (Section A)
- What are the results? (Section B)
- Will the results help locally? (Section C)

The 10 questions on the following pages are designed to help you think about these issues systematically. The first two questions are screening questions and can be answered quickly. If the answer to both is "yes", it is worth proceeding with the remaining questions. There is some degree of overlap between the questions, you are asked to record a "yes", "no" or "can't tell" to most of the questions. A number of italicised prompts are given after each question. These are designed to remind you why the question is important. Record your reasons for your answers in the spaces provided.

About: These checklists were designed to be used as educational pedagogic tools, as part of a workshop setting, therefore we do not suggest a scoring system. The core CASP checklists (randomised controlled trial & systematic review) were based on JAMA 'Users' guides to the medical literature 1994 (adapted from Guyatt GH, Sackett DL, and Cook DJ), and piloted with health care practitioners.

For each new checklist, a group of experts were assembled to develop and pilot the checklist and the workshop format with which it would be used. Over the years overall adjustments have been made to the format, but a recent survey of checklist users reiterated that the basic format continues to be useful and appropriate.

Referencing: we recommend using the Harvard style citation, i.e.: *Critical Appraisal Skills Programme (2018). CASP (insert name of checklist i.e. Qualitative) Checklist. [online] Available at: URL. Accessed: Date Accessed.*

©CASP this work is licensed under the Creative Commons Attribution – Non-Commercial-Share A like. To view a copy of this license, visit http://creativecommons.org/licenses/by-nc-sa/3.0/ www.casp-uk.net

CASP
Critical Appraisal
Skills Programme

Perceived ideal body size of Ghanaian women
— "Not too skinny, but not too fat"

Paper for appraisal and reference:

Section A: Are the results valid?

1. Was there a clear statement of the aims of the research?

Yes	✓
Can't Tell	
No	

HINT: Consider
- what was the goal of the research
- why it was thought important
- its relevance

Comments:

2. Is a qualitative methodology appropriate?

Yes	✓
Can't Tell	
No	

HINT: Consider
- If the research seeks to interpret or illuminate the actions and/or subjective experiences of research participants
- Is qualitative research the right methodology for addressing the research goal

Comments:

Is it worth continuing?

3. Was the research design appropriate to address the aims of the research?

Yes	
Can't Tell	✓
No	

HINT: Consider
- if the researcher has justified the research design (e.g. have they discussed how they decided which method to use)

Comments:

CASP
Critical Appraisal Skills Programme

4. Was the recruitment strategy appropriate to the aims of the research?

Yes	✓
Can't Tell	
No	

HINT: Consider
- If the researcher has explained how the participants were selected
- If they explained why the participants they selected were the most appropriate to provide access to the type of knowledge sought by the study
- If there are any discussions around recruitment (e.g. why some people chose not to take part)

Comments:

5. Was the data collected in a way that addressed the research issue?

Yes	✓
Can't Tell	
No	

HINT: Consider
- If the setting for the data collection was justified
- If it is clear how data were collected (e.g. focus group, semi-structured interview etc.)
- If the researcher has justified the methods chosen
- If the researcher has made the methods explicit (e.g. for interview method, is there an indication of how interviews are conducted, or did they use a topic guide)
- If methods were modified during the study. If so, has the researcher explained how and why
- If the form of data is clear (e.g. tape recordings, video material, notes etc.)
- If the researcher has discussed saturation of data

Comments:

6. Has the relationship between researcher and participants been adequately considered?

Yes	
Can't Tell	
No	✓

HINT: Consider
- If the researcher critically examined their own role, potential bias and influence during (a) formulation of the research questions (b) data collection, including sample recruitment and choice of location
- How the researcher responded to events during the study and whether they considered the implications of any changes in the research design

Comments:

Section B: What are the results?

7. Have ethical issues been taken into consideration?

Yes	✓
Can't Tell	[illegible]
No	

HINT: Consider
- If there are sufficient details of how the research was explained to participants for the reader to assess whether ethical standards were maintained
- If the researcher has discussed issues raised by the study (e.g. issues around informed consent or confidentiality or how they have handled the effects of the study on the participants during and after the study)
- If approval has been sought from the ethics committee

Comments:

8. Was the data analysis sufficiently rigorous?

- [x] Yes
- [ ] Can't Tell
- [ ] No

HINT: Consider
- If there is an in-depth description of the analysis process
- If thematic analysis is used. If so, is it clear how the categories/themes were derived from the data
- Whether the researcher explains how the data presented were selected from the original sample to demonstrate the analysis process
- If sufficient data are presented to support the findings
- To what extent contradictory data are taken into account
- Whether the researcher critically examined their own role, potential bias and influence during analysis and selection of data for presentation

Comments:

9. Is there a clear statement of findings?

- [x] Yes
- [ ] Can't Tell
- [ ] No

HINT: Consider whether
- If the findings are explicit
- If there is adequate discussion of the evidence both for and against the researcher's arguments
- If the researcher has discussed the credibility of their findings (e.g. triangulation, respondent validation, more than one analyst)
- If the findings are discussed in relation to the original research question

Comments:

CASP
Critical Appraisal
Skills Programme

Section C: Will the results help locally?

10. How valuable is the research?

Yes

HINT: Consider
- If the researcher discusses the contribution the study makes to existing knowledge or understanding (e.g. do they consider the findings in relation to current practice or policy, or relevant research-based literature
- If they identify new areas where research is necessary
- If the researchers have discussed whether or how the findings can be transferred to other populations or considered other ways the research may be used

Comments:

Christian [illegible]: Perceived ideal body size of Ghanaian women: "Not too skinny, but not too fat". DONE

CASP
Critical Appraisal Skills Programme

www.casp-uk.net
info@casp-uk.net
Summertown Pavilion, Middle Way Oxford OX2 7LG

CASP Checklist: 10 questions to help you make sense of a **Qualitative** research

How to use this appraisal tool: Three broad issues need to be considered when appraising a qualitative study:

- Are the results of the study valid? (Section A)
- What are the results? (Section B)
- Will the results help locally? (Section C)

The 10 questions on the following pages are designed to help you think about these issues systematically. The first two questions are screening questions and can be answered quickly. If the answer to both is "yes", it is worth proceeding with the remaining questions. There is some degree of overlap between the questions, you are asked to record a "yes", "no" or "can't tell" to most of the questions. A number of italicised prompts are given after each question. These are designed to remind you why the question is important. Record your reasons for your answers in the spaces provided.

About: These checklists were designed to be used as educational pedagogic tools, as part of a workshop setting, therefore we do not suggest a scoring system. The core CASP checklists (randomised controlled trial & systematic review) were based on JAMA 'Users' guides to the medical literature 1994 (adapted from Guyatt GH, Sackett DL, and Cook DJ), and piloted with health care practitioners.

For each new checklist, a group of experts were assembled to develop and pilot the checklist and the workshop format with which it would be used. Over the years overall adjustments have been made to the format, but a recent survey of checklist users reiterated that the basic format continues to be useful and appropriate.

Referencing: we recommend using the Harvard style citation, i.e.: *Critical Appraisal Skills Programme (2018). CASP (insert name of checklist i.e. Qualitative) Checklist. [online] Available at: URL. Accessed: Date Accessed.*

©CASP this work is licensed under the Creative Commons Attribution – Non-Commercial-Share A like. To view a copy of this license, visit http://creativecommons.org/licenses/by-nc-sa/3.0/ www.casp-uk.net

CASP
Critical Appraisal
Skills Programme

Paper for appraisal and reference:

Section A: Are the results valid?

1. Was there a clear statement of the aims of the research?

Yes ☑
Can't Tell ☐
No ☐

HINT: Consider
- what was the goal of the research
- why it was thought important
- its relevance

Comments:

2. Is a qualitative methodology appropriate?

Yes ☑
Can't Tell ☐
No ☐

HINT: Consider
- If the research seeks to interpret or illuminate the actions and/or subjective experiences of research participants
- Is qualitative research the right methodology for addressing the research goal

Comments:

Is it worth continuing?

3. Was the research design appropriate to address the aims of the research?

Yes ☑
Can't Tell ☐
No ☐

HINT: Consider
- if the researcher has justified the research design (e.g. have they discussed how they decided which method to use)

Comments:

4. Was the recruitment strategy appropriate to the aims of the research?	Yes	✓	HINT: Consider
	Can't Tell		• If the researcher has explained how the participants were selected • If they explained why the participants they selected were the most appropriate to provide access to the type of knowledge sought by the study • If there are any discussions around recruitment (e.g. why some people chose not to take part)
	No		

Comments:

5. Was the data collected in a way that addressed the research issue?	Yes	✓	HINT: Consider
	Can't Tell		• If the setting for the data collection was justified • If it is clear how data were collected (e.g. focus group, semi-structured interview etc.) • If the researcher has justified the methods chosen • If the researcher has made the methods explicit (e.g. for interview method, is there an indication of how interviews are conducted, or did they use a topic guide) • If methods were modified during the study. If so, has the researcher explained how and why • If the form of data is clear (e.g. tape recordings, video material, notes etc.) • If the researcher has discussed saturation of data
	No		

Comments:

CASP
Critical Appraisal
Skills Programme

6. Has the relationship between researcher and participants been adequately considered?

Yes	
Can't Tell	✓
No	

HINT: Consider

- If the researcher critically examined their own role, potential bias and influence during (a) formulation of the research questions (b) data collection, including sample recruitment and choice of location
- How the researcher responded to events during the study and whether they considered the implications of any changes in the research design

Comments:

Section B: What are the results?

7. Have ethical issues been taken into consideration?

Yes	✓
Can't Tell	
No	

HINT: Consider

- If there are sufficient details of how the research was explained to participants for the reader to assess whether ethical standards were maintained
- If the researcher has discussed issues raised by the study (e.g. issues around informed consent or confidentiality or how they have handled the effects of the study on the participants during and after the study)
- If approval has been sought from the ethics committee

Comments:

CASP
Critical Appraisal
Skills Programme

8. Was the data analysis sufficiently rigorous?

- Yes ☑
- Can't Tell ☐
- No ☐

HINT: Consider
- If there is an in-depth description of the analysis process
- If thematic analysis is used. If so, is it clear how the categories/themes were derived from the data
- Whether the researcher explains how the data presented were selected from the original sample to demonstrate the analysis process
- If sufficient data are presented to support the findings
- To what extent contradictory data are taken into account
- Whether the researcher critically examined their own role, potential bias and influence during analysis and selection of data for presentation

Comments:

9. Is there a clear statement of findings?

- Yes ☑
- Can't Tell ☐
- No ☐

HINT: Consider whether
- If the findings are explicit
- If there is adequate discussion of the evidence both for and against the researcher's arguments
- If the researcher has discussed the credibility of their findings (e.g. triangulation, respondent validation, more than one analyst)
- If the findings are discussed in relation to the original research question

Comments:

Request for

360 82.7
27
378 1
965

CASP
Critical Appraisal
Skills Programme

Section C: Will the results help locally?

Yes ✓
Can't tell
No

10. How valuable is the research?

HINT: Consider
- If the researcher discusses the contribution the study makes to existing knowledge or understanding (e.g. do they consider the findings in relation to current practice or policy, or relevant research-based literature
- If they identify new areas where research is necessary
- If the researchers have discussed whether or how the findings can be transferred to other populations or considered other ways the research may be used

Comments:

Narratives of urban female adolescents in South Africa: dietary and physical activity practices in an obesogenic environment

CASP
Critical Appraisal Skills Programme

9/16

www.casp-uk.net
info@casp-uk.net
Summertown Pavilion, Middle Way Oxford OX2 7LG

IFEOMA OZODIEGWU

CASP Checklist: 10 questions to help you make sense of a **Qualitative** research

How to use this appraisal tool: Three broad issues need to be considered when appraising a qualitative study:

- Are the results of the study valid? (Section A)
- What are the results? (Section B)
- Will the results help locally? (Section C)

The 10 questions on the following pages are designed to help you think about these issues systematically. The first two questions are screening questions and can be answered quickly. If the answer to both is "yes", it is worth proceeding with the remaining questions. There is some degree of overlap between the questions, you are asked to record a "yes", "no" or "can't tell" to most of the questions. A number of italicised prompts are given after each question. These are designed to remind you why the question is important. Record your reasons for your answers in the spaces provided.

About: These checklists were designed to be used as educational pedagogic tools, as part of a workshop setting, therefore we do not suggest a scoring system. The core CASP checklists (randomised controlled trial & systematic review) were based on JAMA 'Users' guides to the medical literature 1994 (adapted from Guyatt GH, Sackett DL, and Cook DJ), and piloted with health care practitioners.

For each new checklist, a group of experts were assembled to develop and pilot the checklist and the workshop format with which it would be used. Over the years overall adjustments have been made to the format, but a recent survey of checklist users reiterated that the basic format continues to be useful and appropriate.

Referencing: we recommend using the Harvard style citation, i.e.: *Critical Appraisal Skills Programme (2018). CASP (insert name of checklist i.e. Qualitative) Checklist. [online] Available at: URL. Accessed: Date Accessed.*

©CASP this work is licensed under the Creative Commons Attribution – Non-Commercial-Share A like. To view a copy of this license, visit http://creativecommons.org/licenses/by-nc-sa/3.0/ www.casp-uk.net

CASP
Critical Appraisal Skills Programme

Paper for appraisal and reference:

Section A: Are the results valid?

1. Was there a clear statement of the aims of the research?

Yes ✓
Can't Tell
No

HINT: Consider
- what was the goal of the research
- why it was thought important
- its relevance

Comments:

2. Is a qualitative methodology appropriate?

Yes ✓
Can't Tell
No

HINT: Consider
- If the research seeks to interpret or illuminate the actions and/or subjective experiences of research participants
- Is qualitative research the right methodology for addressing the research goal

Comments:

Is it worth continuing?

3. Was the research design appropriate to address the aims of the research?

Yes ✓
Can't Tell
No

HINT: Consider
- if the researcher has justified the research design (e.g. have they discussed how they decided which method to use)

Comments:

CASP
Critical Appraisal Skills Programme

4. Was the recruitment strategy appropriate to the aims of the research?		HINT: Consider
	Yes ✓	• If the researcher has explained how the participants were selected
	Can't Tell ☐	• If they explained why the participants they selected were the most appropriate to provide access to the type of knowledge sought by the study
	No ☐	• If there are any discussions around recruitment (e.g. why some people chose not to take part)

Comments:

5. Was the data collected in a way that addressed the research issue?		HINT: Consider
	Yes ✓	• If the setting for the data collection was justified
	Can't Tell ☐	• If it is clear how data were collected (e.g. focus group, semi-structured interview etc.)
	No ☐	• If the researcher has justified the methods chosen
		• If the researcher has made the methods explicit (e.g. for interview method, is there an indication of how interviews are conducted, or did they use a topic guide)
		• If methods were modified during the study. If so, has the researcher explained how and why
		• If the form of data is clear (e.g. tape recordings, video material, notes etc.)
		• If the researcher has discussed saturation of data

Comments:

Critical Appraisal Skills Programme

6. Has the relationship between researcher and participants been adequately considered?

Yes	
Can't Tell	
No	✓

HINT: Consider
- If the researcher critically examined their own role, potential bias and influence during (a) formulation of the research questions (b) data collection, including sample recruitment and choice of location
- How the researcher responded to events during the study and whether they considered the implications of any changes in the research design

Comments:

Section B: What are the results?

7. Have ethical issues been taken into consideration?

Yes	✓
Can't Tell	
No	

HINT: Consider
- If there are sufficient details of how the research was explained to participants for the reader to assess whether ethical standards were maintained
- If the researcher has discussed issues raised by the study (e.g. issues around informed consent or confidentiality or how they have handled the effects of the study on the participants during and after the study)
- If approval has been sought from the ethics committee

Comments:

CASP
Critical Appraisal Skills Programme

8. Was the data analysis sufficiently rigorous?

Yes	✓
Can't Tell	
No	

HINT: Consider
- If there is an in-depth description of the analysis process
- If thematic analysis is used. If so, is it clear how the categories/themes were derived from the data
- Whether the researcher explains how the data presented were selected from the original sample to demonstrate the analysis process
- If sufficient data are presented to support the findings
- To what extent contradictory data are taken into account
- Whether the researcher critically examined their own role, potential bias and influence during analysis and selection of data for presentation

Comments:

9. Is there a clear statement of findings?

Yes	✓
Can't Tell	
No	

HINT: Consider whether
- If the findings are explicit
- If there is adequate discussion of the evidence both for and against the researcher's arguments
- If the researcher has discussed the credibility of their findings (e.g. triangulation, respondent validation, more than one analyst)
- If the findings are discussed in relation to the original research question

Comments:

CASP
Critical Appraisal
Skills Programme

Section C: Will the results help locally?

10. How valuable is the research?

Yes

HINT: Consider
- If the researcher discusses the contribution the study makes to existing knowledge or understanding (e.g. do they consider the findings in relation to current practice or policy, or relevant research-based literature
- If they identify new areas where research is necessary
- If the researchers have discussed whether or how the findings can be transferred to other populations or considered other ways the research may be used

Comments:

Christian Nwabueze: Narratives of urban female adolescents in South Africa: dietary and physical activity practices in an obesogenic environment.

CASP
Critical Appraisal Skills Programme

www.casp-uk.net
info@casp-uk.net
Summertown Pavilion, Middle Way Oxford OX2 7LG

CASP Checklist: 10 questions to help you make sense of a **Qualitative** research

How to use this appraisal tool: Three broad issues need to be considered when appraising a qualitative study:

- Are the results of the study valid? (Section A)
- What are the results? (Section B)
- Will the results help locally? (Section C)

The 10 questions on the following pages are designed to help you think about these issues systematically. The first two questions are screening questions and can be answered quickly. If the answer to both is "yes", it is worth proceeding with the remaining questions. There is some degree of overlap between the questions, you are asked to record a "yes", "no" or "can't tell" to most of the questions. A number of italicised prompts are given after each question. These are designed to remind you why the question is important. Record your reasons for your answers in the spaces provided.

About: These checklists were designed to be used as educational pedagogic tools, as part of a workshop setting, therefore we do not suggest a scoring system. The core CASP checklists (randomised controlled trial & systematic review) were based on JAMA 'Users' guides to the medical literature 1994 (adapted from Guyatt GH, Sackett DL, and Cook DJ), and piloted with health care practitioners.

For each new checklist, a group of experts were assembled to develop and pilot the checklist and the workshop format with which it would be used. Over the years overall adjustments have been made to the format, but a recent survey of checklist users reiterated that the basic format continues to be useful and appropriate.

Referencing: we recommend using the Harvard style citation, i.e.: *Critical Appraisal Skills Programme (2018). CASP (insert name of checklist i.e. Qualitative) Checklist. [online] Available at: URL. Accessed: Date Accessed.*

©CASP this work is licensed under the Creative Commons Attribution – Non-Commercial-Share A like. To view a copy of this license, visit http://creativecommons.org/licenses/by-nc-sa/3.0/ www.casp-uk.net

CASP
Critical Appraisal Skills Programme

Paper for appraisal and reference:

Section A: Are the results valid?

1. Was there a clear statement of the aims of the research?

Yes ✓ | Can't Tell | No

HINT: Consider
- what was the goal of the research
- why it was thought important
- its relevance

Comments:

2. Is a qualitative methodology appropriate?

Yes ✓ | Can't Tell | No

HINT: Consider
- If the research seeks to interpret or illuminate the actions and/or subjective experiences of research participants
- Is qualitative research the right methodology for addressing the research goal

Comments:

Is it worth continuing?

3. Was the research design appropriate to address the aims of the research?

Yes ✓ | Can't Tell | No

HINT: Consider
- if the researcher has justified the research design (e.g. have they discussed how they decided which method to use)

Comments:

4. Was the recruitment strategy appropriate to the aims of the research?	Yes	✓	HINT: Consider<br>• If the researcher has explained how the participants were selected<br>• If they explained why the participants they selected were the most appropriate to provide access to the type of knowledge sought by the study<br>• If there are any discussions around recruitment (e.g. why some people chose not to take part)
	Can't Tell		
	No		

Comments:

5. Was the data collected in a way that addressed the research issue?	Yes	✓	HINT: Consider<br>• If the setting for the data collection was justified<br>• If it is clear how data were collected (e.g. focus group, semi-structured interview etc.)<br>• If the researcher has justified the methods chosen<br>• If the researcher has made the methods explicit (e.g. for interview method, is there an indication of how interviews are conducted, or did they use a topic guide)<br>• If methods were modified during the study. If so, has the researcher explained how and why<br>• If the form of data is clear (e.g. tape recordings, video material, notes etc.)<br>• If the researcher has discussed saturation of data
	Can't Tell		
	No		

Comments:

CASP
Critical Appraisal Skills Programme

6. Has the relationship between researcher and participants been adequately considered?	Yes		HINT: Consider<br>• If the researcher critically examined their own role, potential bias and influence during (a) formulation of the research questions (b) data collection, including sample recruitment and choice of location<br>• How the researcher responded to events during the study and whether they considered the implications of any changes in the research design
	Can't Tell		
	No		

Comments:

Section B: What are the results?

7. Have ethical issues been taken into consideration?	Yes		HINT: Consider<br>• If there are sufficient details of how the research was explained to participants for the reader to assess whether ethical standards were maintained<br>• If the researcher has discussed issues raised by the study (e.g. issues around informed consent or confidentiality or how they have handled the effects of the study on the participants during and after the study)<br>• If approval has been sought from the ethics committee
	Can't Tell		
	No		

Comments:

CASP
Critical Appraisal
Skills Programme

8. Was the data analysis sufficiently rigorous?

Yes	✓
Can't Tell	
No	

HINT: Consider
- If there is an in-depth description of the analysis process
- If thematic analysis is used. If so, is it clear how the categories/themes were derived from the data
- Whether the researcher explains how the data presented were selected from the original sample to demonstrate the analysis process
- If sufficient data are presented to support the findings
- To what extent contradictory data are taken into account
- Whether the researcher critically examined their own role, potential bias and influence during analysis and selection of data for presentation

Comments:

9. Is there a clear statement of findings?

Yes	✓
Can't Tell	
No	

HINT: Consider whether
- If the findings are explicit
- If there is adequate discussion of the evidence both for and against the researcher's arguments
- If the researcher has discussed the credibility of their findings (e.g. triangulation, respondent validation, more than one analyst)
- If the findings are discussed in relation to the original research question

Comments:

CASP
Critical Appraisal
Skills Programme

Section C: Will the results help locally?

Yes ✓

10. How valuable is the research?

Can't tell

No

HINT: Consider
- If the researcher discusses the contribution the study makes to existing knowledge or understanding (e.g. do they consider the findings in relation to current practice or policy, or relevant research-based literature
- If they identify new areas where research is necessary
- If the researchers have discussed whether or how the findings can be transferred to other populations or considered other ways the research may be used

Comments:

Perceptions of diet, physical activity, and obesity-related health among black daughter-mother pairs in Soweto, South Africa: a qualitative study

CASP

Critical Appraisal Skills Programme

www.casp-uk.net

info@casp-uk.net

Summertown Pavilion, Middle Way Oxford OX2 7LG

9/10

IFEOMA OZODIEGWU

CASP Checklist: 10 questions to help you make sense of a **Qualitative** research

How to use this appraisal tool: Three broad issues need to be considered when appraising a qualitative study:

- Are the results of the study valid? (Section A)
- What are the results? (Section B)
- Will the results help locally? (Section C)

The 10 questions on the following pages are designed to help you think about these issues systematically. The first two questions are screening questions and can be answered quickly. If the answer to both is "yes", it is worth proceeding with the remaining questions. There is some degree of overlap between the questions, you are asked to record a "yes", "no" or "can't tell" to most of the questions. A number of italicised prompts are given after each question. These are designed to remind you why the question is important. Record your reasons for your answers in the spaces provided.

About: These checklists were designed to be used as educational pedagogic tools, as part of a workshop setting, therefore we do not suggest a scoring system. The core CASP checklists (randomised controlled trial & systematic review) were based on JAMA 'Users' guides to the medical literature 1994 (adapted from Guyatt GH, Sackett DL, and Cook DJ), and piloted with health care practitioners.

For each new checklist, a group of experts were assembled to develop and pilot the checklist and the workshop format with which it would be used. Over the years overall adjustments have been made to the format, but a recent survey of checklist users reiterated that the basic format continues to be useful and appropriate.

Referencing: we recommend using the Harvard style citation, i.e.: *Critical Appraisal Skills Programme (2018). CASP (insert name of checklist i.e. Qualitative) Checklist. [online] Available at: URL. Accessed: Date Accessed.*

©CASP this work is licensed under the Creative Commons Attribution – Non-Commercial-Share A like. To view a copy of this license, visit http://creativecommons.org/licenses/by-nc-sa/3.0/ www.casp-uk.net

CASP
Critical Appraisal
Skills Programme

Perceptions of diet, physical activity, and obesity-related health among black daughter-mother pairs in Soweto, SA: a qualitative study.

Paper for appraisal and reference:

Section A: Are the results valid?

1. Was there a clear statement of the aims of the research?

- Yes ✓
- Can't Tell
- No

HINT: Consider
- what was the goal of the research
- why it was thought important
- its relevance

Comments:

2. Is a qualitative methodology appropriate?

- Yes ✓
- Can't Tell
- No

HINT: Consider
- If the research seeks to interpret or illuminate the actions and/or subjective experiences of research participants
- Is qualitative research the right methodology for addressing the research goal

Comments:

Is it worth continuing?

3. Was the research design appropriate to address the aims of the research?

- Yes ✓
- Can't Tell
- No

HINT: Consider
- if the researcher has justified the research design (e.g. have they discussed how they decided which method to use)

Comments:

CASP
Critical Appraisal
Skills Programme

Perceptions of diet, physical activity, and obesity-related health among black daughter-mother pairs in Soweto, SA: a qualitative study.

Paper for appraisal and reference:

Section A: Are the results valid?

1. Was there a clear statement of the aims of the research?

- Yes ✓
- Can't Tell
- No

HINT: Consider
- what was the goal of the research
- why it was thought important
- its relevance

Comments:

2. Is a qualitative methodology appropriate?

- Yes ✓
- Can't Tell
- No

HINT: Consider
- If the research seeks to interpret or illuminate the actions and/or subjective experiences of research participants
- Is qualitative research the right methodology for addressing the research goal

Comments:

Is it worth continuing?

3. Was the research design appropriate to address the aims of the research?

- Yes ✓
- Can't Tell
- No

HINT: Consider
- if the researcher has justified the research design (e.g. have they discussed how they decided which method to use)

Comments:

CASP
Critical Appraisal Skills Programme

4. Was the recruitment strategy appropriate to the aims of the research?	Yes ✓<br>Can't Tell<br>No	HINT: Consider<br>• If the researcher has explained how the participants were selected<br>• If they explained why the participants they selected were the most appropriate to provide access to the type of knowledge sought by the study<br>• If there are any discussions around recruitment (e.g. why some people chose not to take part)

Comments:

5. Was the data collected in a way that addressed the research issue?	Yes ✓<br>Can't Tell<br>No	HINT: Consider<br>• If the setting for the data collection was justified<br>• If it is clear how data were collected (e.g. focus group, semi-structured interview etc.)<br>• If the researcher has justified the methods chosen<br>• If the researcher has made the methods explicit (e.g. for interview method, is there an indication of how interviews are conducted, or did they use a topic guide)<br>• If methods were modified during the study. If so, has the researcher explained how and why<br>• If the form of data is clear (e.g. tape recordings, video material, notes etc.)<br>• If the researcher has discussed saturation of data

Comments:

8. Was the data analysis sufficiently rigorous?

Yes ☑
Can't Tell ☐
No ☐

HINT: Consider
- If there is an in-depth description of the analysis process
- If thematic analysis is used. If so, is it clear how the categories/themes were derived from the data
- Whether the researcher explains how the data presented were selected from the original sample to demonstrate the analysis process
- If sufficient data are presented to support the findings
- To what extent contradictory data are taken into account
- Whether the researcher critically examined their own role, potential bias and influence during analysis and selection of data for presentation

Comments:

9. Is there a clear statement of findings?

Yes ☑
Can't Tell ☐
No ☐

HINT: Consider whether
- If the findings are explicit
- If there is adequate discussion of the evidence both for and against the researcher's arguments
- If the researcher has discussed the credibility of their findings (e.g. triangulation, respondent validation, more than one analyst)
- If the findings are discussed in relation to the original research question

Comments:

C∧SP
Critical Appraisal
Skills Programme

Section C: Will the results help locally?

10. How valuable is the research?

Yes

HINT: Consider
- If the researcher discusses the contribution the study makes to existing knowledge or understanding (e.g. do they consider the findings in relation to current practice or policy, or relevant research-based literature
- If they identify new areas where research is necessary
- If the researchers have discussed whether or how the findings can be transferred to other populations or considered other ways the research may be used

Comments:

Chastan Nwabueze: Perceptions of diet, physical activity, and obesity-related health among black daughter-mother pairs in Soweto, South Africa: a qualitative study

CASP
Critical Appraisal
Skills Programme

www.casp-uk.net
info@casp-uk.net
Summertown Pavilion, Middle Way Oxford OX2 7LG

CASP Checklist: 10 questions to help you make sense of a **Qualitative** research

How to use this appraisal tool: Three broad issues need to be considered when appraising a qualitative study:

- Are the results of the study valid? (Section A)
- What are the results? (Section B)
- Will the results help locally? (Section C)

The 10 questions on the following pages are designed to help you think about these issues systematically. The first two questions are screening questions and can be answered quickly. If the answer to both is "yes", it is worth proceeding with the remaining questions. There is some degree of overlap between the questions, you are asked to record a "yes", "no" or "can't tell" to most of the questions. A number of italicised prompts are given after each question. These are designed to remind you why the question is important. Record your reasons for your answers in the spaces provided.

About: These checklists were designed to be used as educational pedagogic tools, as part of a workshop setting, therefore we do not suggest a scoring system. The core CASP checklists (randomised controlled trial & systematic review) were based on JAMA 'Users' guides to the medical literature 1994 (adapted from Guyatt GH, Sackett DL, and Cook DJ), and piloted with health care practitioners.

For each new checklist, a group of experts were assembled to develop and pilot the checklist and the workshop format with which it would be used. Over the years overall adjustments have been made to the format, but a recent survey of checklist users reiterated that the basic format continues to be useful and appropriate.

Referencing: we recommend using the Harvard style citation, i.e.: *Critical Appraisal Skills Programme (2018). CASP (insert name of checklist i.e. Qualitative) Checklist. [online] Available at: URL. Accessed: Date Accessed.*

©CASP this work is licensed under the Creative Commons Attribution – Non-Commercial-Share A like. To view a copy of this license, visit http://creativecommons.org/licenses/by-nc-sa/3.0/ www.casp-uk.net

CASP
Critical Appraisal
Skills Programme

Paper for appraisal and reference:

Section A: Are the results valid?

1. Was there a clear statement of the aims of the research?

Yes ✓
Can't Tell
No

HINT: Consider
- what was the goal of the research
- why it was thought important
- its relevance

Comments:

2. Is a qualitative methodology appropriate?

Yes ✓
Can't Tell
No

HINT: Consider
- If the research seeks to interpret or illuminate the actions and/or subjective experiences of research participants
- Is qualitative research the right methodology for addressing the research goal

Comments:

Is it worth continuing?

3. Was the research design appropriate to address the aims of the research?

Yes ✓
Can't Tell
No

HINT: Consider
- if the researcher has justified the research design (e.g. have they discussed how they decided which method to use)

Comments:

4. Was the recruitment strategy appropriate to the aims of the research?	Yes	✓	HINT: Consider<br>• If the researcher has explained how the participants were selected<br>• If they explained why the participants they selected were the most appropriate to provide access to the type of knowledge sought by the study<br>• If there are any discussions around recruitment (e.g. why some people chose not to take part)
	Can't Tell		
	No		

Comments:

5. Was the data collected in a way that addressed the research issue?	Yes	✓	HINT: Consider<br>• If the setting for the data collection was justified<br>• If it is clear how data were collected (e.g. focus group, semi-structured interview etc.)<br>• If the researcher has justified the methods chosen<br>• If the researcher has made the methods explicit (e.g. for interview method, is there an indication of how interviews are conducted, or did they use a topic guide)<br>• If methods were modified during the study. If so, has the researcher explained how and why<br>• If the form of data is clear (e.g. tape recordings, video material, notes etc.)<br>• If the researcher has discussed saturation of data
	Can't Tell		
	No		

Comments:

CASP
Critical Appraisal
Skills Programme

6. Has the relationship between researcher and participants been adequately considered?

Yes [ ]
Can't Tell [ ]
No [ ]

HINT: Consider
- If the researcher critically examined their own role, potential bias and influence during (a) formulation of the research questions (b) data collection, including sample recruitment and choice of location
- How the researcher responded to events during the study and whether they considered the implications of any changes in the research design

Comments:

Section B: What are the results?

7. Have ethical issues been taken into consideration?

Yes [✓]
Can't Tell [ ]
No [ ]

HINT: Consider
- If there are sufficient details of how the research was explained to participants for the reader to assess whether ethical standards were maintained
- If the researcher has discussed issues raised by the study (e.g. issues around informed consent or confidentiality or how they have handled the effects of the study on the participants during and after the study)
- If approval has been sought from the ethics committee

Comments:

CASP
Critical Appraisal
Skills Programme

8. Was the data analysis sufficiently rigorous?	Yes	✓	HINT: Consider<br>• If there is an in-depth description of the analysis process<br>• If thematic analysis is used. If so, is it clear how the categories/themes were derived from the data<br>• Whether the researcher explains how the data presented were selected from the original sample to demonstrate the analysis process<br>• If sufficient data are presented to support the findings<br>• To what extent contradictory data are taken into account<br>• Whether the researcher critically examined their own role, potential bias and influence during analysis and selection of data for presentation
	Can't Tell		
	No		

Comments:

9. Is there a clear statement of findings?	Yes	✓	HINT: Consider whether<br>• If the findings are explicit<br>• If there is adequate discussion of the evidence both for and against the researcher's arguments<br>• If the researcher has discussed the credibility of their findings (e.g. triangulation, respondent validation, more than one analyst)<br>• If the findings are discussed in relation to the original research question
	Can't Tell		
	No		

Comments:

CASP
Critical Appraisal Skills Programme

Section C: Will the results help locally?

10. How valuable is the research?

HINT: Consider
- If the researcher discusses the contribution the study makes to existing knowledge or understanding (e.g. do they consider the findings in relation to current practice or policy, or relevant research-based literature
- If they identify new areas where research is necessary
- If the researchers have discussed whether or how the findings can be transferred to other populations or considered other ways the research may be used

Comments:

'We eat together, to say she buys, tomorrow I will buy the food': adolescent best friends' food choices and dietary practices in Soweto, South Africa

9/10

CASP
Critical Appraisal Skills Programme

www.casp-uk.net

info@casp-uk.net

Summertown Pavilion, Middle Way Oxford OX2 7LG

IFEOMA OZODIEGWU

CASP Checklist: 10 questions to help you make sense of a **Qualitative** research

How to use this appraisal tool: Three broad issues need to be considered when appraising a qualitative study:

- Are the results of the study valid? (Section A)
- What are the results? (Section B)
- Will the results help locally? (Section C)

The 10 questions on the following pages are designed to help you think about these issues systematically. The first two questions are screening questions and can be answered quickly. If the answer to both is "yes", it is worth proceeding with the remaining questions. There is some degree of overlap between the questions, you are asked to record a "yes", "no" or "can't tell" to most of the questions. A number of italicised prompts are given after each question. These are designed to remind you why the question is important. Record your reasons for your answers in the spaces provided.

About: These checklists were designed to be used as educational pedagogic tools, as part of a workshop setting, therefore we do not suggest a scoring system. The core CASP checklists (randomised controlled trial & systematic review) were based on JAMA 'Users' guides to the medical literature 1994 (adapted from Guyatt GH, Sackett DL, and Cook DJ), and piloted with health care practitioners.

For each new checklist, a group of experts were assembled to develop and pilot the checklist and the workshop format with which it would be used. Over the years overall adjustments have been made to the format, but a recent survey of checklist users reiterated that the basic format continues to be useful and appropriate.

Referencing: we recommend using the Harvard style citation, i.e.: *Critical Appraisal Skills Programme (2018). CASP (insert name of checklist i.e. Qualitative) Checklist. [online] Available at: URL. Accessed: Date Accessed.*

©CASP this work is licensed under the Creative Commons Attribution – Non-Commercial-Share A like. To view a copy of this license, visit http://creativecommons.org/licenses/by-nc-sa/3.0/ www.casp-uk.net

CASP
Critical Appraisal
Skills Programme

Paper for appraisal and reference:

Section A: Are the results valid?

1. Was there a clear statement of the aims of the research?

Yes ✓ | Can't Tell | No

HINT: Consider
- what was the goal of the research
- why it was thought important
- its relevance

Comments:

2. Is a qualitative methodology appropriate?

Yes ✓ | Can't Tell | No

HINT: Consider
- If the research seeks to interpret or illuminate the actions and/or subjective experiences of research participants
- Is qualitative research the right methodology for addressing the research goal

Comments:

Is it worth continuing?

3. Was the research design appropriate to address the aims of the research?

Yes ✓ | Can't Tell | No

HINT: Consider
- if the researcher has justified the research design (e.g. have they discussed how they decided which method to use)

Comments:

4. Was the recruitment strategy appropriate to the aims of the research?

Yes	✓
Can't Tell	
No	

HINT: Consider
- If the researcher has explained how the participants were selected
- If they explained why the participants they selected were the most appropriate to provide access to the type of knowledge sought by the study
- If there are any discussions around recruitment (e.g. why some people chose not to take part)

Comments:

5. Was the data collected in a way that addressed the research issue?

Yes	✓
Can't Tell	
No	

HINT: Consider
- If the setting for the data collection was justified
- If it is clear how data were collected (e.g. focus group, semi-structured interview etc.)
- If the researcher has justified the methods chosen
- If the researcher has made the methods explicit (e.g. for interview method, is there an indication of how interviews are conducted, or did they use a topic guide)
- If methods were modified during the study. If so, has the researcher explained how and why
- If the form of data is clear (e.g. tape recordings, video material, notes etc.)
- If the researcher has discussed saturation of data

Comments:

6. Has the relationship between researcher and participants been adequately considered?

Yes	
Can't Tell	
No	✓

HINT: Consider

- If the researcher critically examined their own role, potential bias and influence during (a) formulation of the research questions (b) data collection, including sample recruitment and choice of location
- How the researcher responded to events during the study and whether they considered the implications of any changes in the research design

Comments:

Section B: What are the results?

7. Have ethical issues been taken into consideration?

Yes	✓
Can't Tell	[illegible]
No	

HINT: Consider

- If there are sufficient details of how the research was explained to participants for the reader to assess whether ethical standards were maintained
- If the researcher has discussed issues raised by the study (e.g. issues around informed consent or confidentiality or how they have handled the effects of the study on the participants during and after the study)
- If approval has been sought from the ethics committee

Comments:

CASP
Critical Appraisal Skills Programme

8. Was the data analysis sufficiently rigorous?

Yes	✓
Can't Tell	
No	

HINT: Consider
- If there is an in-depth description of the analysis process
- If thematic analysis is used. If so, is it clear how the categories/themes were derived from the data
- Whether the researcher explains how the data presented were selected from the original sample to demonstrate the analysis process
- If sufficient data are presented to support the findings
- To what extent contradictory data are taken into account
- Whether the researcher critically examined their own role, potential bias and influence during analysis and selection of data for presentation

Comments:

9. Is there a clear statement of findings?

Yes	✓
Can't Tell	
No	

HINT: Consider whether
- If the findings are explicit
- If there is adequate discussion of the evidence both for and against the researcher's arguments
- If the researcher has discussed the credibility of their findings (e.g. triangulation, respondent validation, more than one analyst)
- If the findings are discussed in relation to the original research question

Comments:

Section C: Will the results help locally?

10. How valuable is the research?

Yes

HINT: Consider
- If the researcher discusses the contribution the study makes to existing knowledge or understanding (e.g. do they consider the findings in relation to current practice or policy, or relevant research-based literature
- If they identify new areas where research is necessary
- If the researchers have discussed whether or how the findings can be transferred to other populations or considered other ways the research may be used

Comments:

Christian [illegible]: "We eat together, today she buys, tomorrow I will buy the food!": adolescent best friends' food choices and dietary practices in Soweto, South Africa DONE

CASP
Critical Appraisal Skills Programme

www.casp-uk.net

info@casp-uk.net

Summertown Pavilion, Middle Way Oxford OX2 7LG

CASP Checklist: 10 questions to help you make sense of a **Qualitative** research

How to use this appraisal tool: Three broad issues need to be considered when appraising a qualitative study:

- Are the results of the study valid? (Section A)
- What are the results? (Section B)
- Will the results help locally? (Section C)

The 10 questions on the following pages are designed to help you think about these issues systematically. The first two questions are screening questions and can be answered quickly. If the answer to both is "yes", it is worth proceeding with the remaining questions. There is some degree of overlap between the questions, you are asked to record a "yes", "no" or "can't tell" to most of the questions. A number of italicised prompts are given after each question. These are designed to remind you why the question is important. Record your reasons for your answers in the spaces provided.

About: These checklists were designed to be used as educational pedagogic tools, as part of a workshop setting, therefore we do not suggest a scoring system. The core CASP checklists (randomised controlled trial & systematic review) were based on JAMA 'Users' guides to the medical literature 1994 (adapted from Guyatt GH, Sackett DL, and Cook DJ), and piloted with health care practitioners.

For each new checklist, a group of experts were assembled to develop and pilot the checklist and the workshop format with which it would be used. Over the years overall adjustments have been made to the format, but a recent survey of checklist users reiterated that the basic format continues to be useful and appropriate.

Referencing: we recommend using the Harvard style citation, i.e.: *Critical Appraisal Skills Programme (2018). CASP (insert name of checklist i.e. Qualitative) Checklist. [online] Available at: URL. Accessed: Date Accessed.*

©CASP this work is licensed under the Creative Commons Attribution – Non-Commercial-Share A like. To view a copy of this license, visit http://creativecommons.org/licenses/by-nc-sa/3.0/ www.casp-uk.net

CASP
Critical Appraisal Skills Programme

Paper for appraisal and reference:

Section A: Are the results valid?

1. Was there a clear statement of the aims of the research?

Yes	✓
Can't Tell	
No	

HINT: Consider
- what was the goal of the research
- why it was thought important
- its relevance

Comments:

2. Is a qualitative methodology appropriate?

Yes	✓
Can't Tell	
No	

HINT: Consider
- If the research seeks to interpret or illuminate the actions and/or subjective experiences of research participants
- Is qualitative research the right methodology for addressing the research goal

Comments:

Is it worth continuing?

3. Was the research design appropriate to address the aims of the research?

Yes	✓
Can't Tell	
No	

HINT: Consider
- if the researcher has justified the research design (e.g. have they discussed how they decided which method to use)

Comments:

4. Was the recruitment strategy appropriate to the aims of the research?

Yes	✓
Can't Tell	
No	

HINT: Consider
- If the researcher has explained how the participants were selected
- If they explained why the participants they selected were the most appropriate to provide access to the type of knowledge sought by the study
- If there are any discussions around recruitment (e.g. why some people chose not to take part)

Comments:

5. Was the data collected in a way that addressed the research issue?

Yes	✓
Can't Tell	
No	

HINT: Consider
- If the setting for the data collection was justified
- If it is clear how data were collected (e.g. focus group, semi-structured interview etc.)
- If the researcher has justified the methods chosen
- If the researcher has made the methods explicit (e.g. for interview method, is there an indication of how interviews are conducted, or did they use a topic guide)
- If methods were modified during the study. If so, has the researcher explained how and why
- If the form of data is clear (e.g. tape recordings, video material, notes etc.)
- If the researcher has discussed saturation of data

Comments:

6. Has the relationship between researcher and participants been adequately considered?

Yes	
Can't Tell	
No	✓

HINT: Consider
- If the researcher critically examined their own role, potential bias and influence during (a) formulation of the research questions (b) data collection, including sample recruitment and choice of location
- How the researcher responded to events during the study and whether they considered the implications of any changes in the research design

Comments:

Section B: What are the results?

7. Have ethical issues been taken into consideration?

Yes	✓
Can't Tell	
No	

HINT: Consider
- If there are sufficient details of how the research was explained to participants for the reader to assess whether ethical standards were maintained
- If the researcher has discussed issues raised by the study (e.g. issues around informed consent or confidentiality or how they have handled the effects of the study on the participants during and after the study)
- If approval has been sought from the ethics committee

Comments:

CASP
Critical Appraisal
Skills Programme

8. Was the data analysis sufficiently rigorous?

Yes	✓
Can't Tell	
No	

HINT: Consider
- If there is an in-depth description of the analysis process
- If thematic analysis is used. If so, is it clear how the categories/themes were derived from the data
- Whether the researcher explains how the data presented were selected from the original sample to demonstrate the analysis process
- If sufficient data are presented to support the findings
- To what extent contradictory data are taken into account
- Whether the researcher critically examined their own role, potential bias and influence during analysis and selection of data for presentation

Comments:

9. Is there a clear statement of findings?

Yes	✓
Can't Tell	
No	

HINT: Consider whether
- If the findings are explicit
- If there is adequate discussion of the evidence both for and against the researcher's arguments
- If the researcher has discussed the credibility of their findings (e.g. triangulation, respondent validation, more than one analyst)
- If the findings are discussed in relation to the original research question

Comments:

CASP
Critical Appraisal
Skills Programme

Section C: Will the results help locally?

10. How valuable is the research?

Yes

Can't Tell

No

HINT: Consider

- If the researcher discusses the contribution the study makes to existing knowledge or understanding (e.g. do they consider the findings in relation to current practice or policy, or relevant research-based literature
- If they identify new areas where research is necessary
- If the researchers have discussed whether or how the findings can be transferred to other populations or considered other ways the research may be used

Comments:
